# Diffuse water pollution during recent extreme wet-weather in the UK: Environmental damage costs and insight into the future?

**DOI:** 10.1016/j.jclepro.2022.130633

**Published:** 2022-03-01

**Authors:** Y. Zhang, S.J. Granger, M.A. Semenov, H.R. Upadhayay, A.L. Collins

**Affiliations:** a Sustainable Agriculture Sciences, Rothamsted Research, North Wyke, Okehampton, EX20 2SB, UK; b Plant Sciences Department, Rothamsted Research, West Common, Harpenden, AL5 2JQ, UK

**Keywords:** Wet-weather, Climate change, Water quality, Nitrate, Suspended sediment

## Abstract

Periods of extreme wet-weather elevate agricultural diffuse water pollutant loads and climate projections for the UK suggest wetter winters. Within this context, we monitored nitrate and suspended sediment loss using a field and landscape scale platform in SW England during the recent extreme wet-weather of 2019–2020. We compared the recent extreme wet-weather period to both the climatic baseline (1981–2010) and projected near- (2041–2060) and far- (2071–2090) future climates, using the 95th percentiles of conventional rainfall indices generated for climate scenarios downscaled by the LARS-WG weather generator from the 19 global climate models in the CMIP5 ensemble for the RCP8.5 emission scenario. Finally, we explored relationships between pollutant loss and the rainfall indices. Grassland field-scale monthly average nitrate losses increased from 0.39-1.07 kg ha^−1^ (2016–2019) to 0.70–1.35 kg ha^−1^ (2019–2020), whereas losses from grassland ploughed up for cereals, increased from 0.63-0.83 kg ha^−1^ to 2.34–4.09 kg ha^−1^. Nitrate losses at landscape scale increased during the 2019–2020 extreme wet-weather period to 2.04–4.54 kg ha^−1^. Field-scale grassland monthly average sediment losses increased from 92-116 kg ha^−1^ (2016–2019) to 281–333 kg ha^−1^ (2019–2020), whereas corresponding losses from grassland converted to cereal production increased from 63-80 kg ha^−1^ to 2124–2146 kg ha^−1^. Landscape scale monthly sediment losses increased from 8-37 kg ha^−1^ in 2018 to between 15 and 173 kg ha^−1^ during the 2019–2020 wet-weather period. 2019–2020 was most representative of the forecast 95th percentiles of >1 mm rainfall for near- and far-future climates and this rainfall index was related to monitored sediment, but not nitrate, loss. The elevated suspended sediment loads generated by the extreme wet-weather of 2019–2020 therefore potentially provide some insight into the responses to the projected >1 mm rainfall extremes under future climates at the study location.

## Highlights

• Rainfall is a key driver of agricultural externalities on water.
• Field - landscape scale pollution emissions were elevated in an extreme wet period.
• Rainfall indices were used to compare the wet period with projected future climates.
• 95th percentiles of >1 mm rainfall are comparable between the present and future.
• Monitored elevated sediment loss was driven by the extremes of >1 mm rainfall.

## 1 Introduction

Water quality faces threats globally from both climate change and intensive farming (Dunn et al., 2012; Michalak, 2016; Malhi et al., 2020). Managing land to produce food whilst ensuring clean surface and ground water for the environment and society continues to be a demanding challenge (Grafton et al., 2015) and contemporary farming remains a significant source of water pollution, including that arising from nitrate and sediment, across scales (Zhang et al., 2014). Agriculture is heavily dependent on environmental conditions, and especially weather patterns, for its productivity and profitability (Harkness et al., 2020). Interactions between weather patterns, climate change and agriculture impact water quality, aquatic ecosystems and water availability (Whitehead et al., 2009; Arnell et al., 2015). The climate-land-water nexus is important since river systems are among the ecosystems most sensitive to climate change (Millennium Ecosystem Assessment, 2005; Watts et al., 2015). Understanding the implications of climate change, weather extremes and land use in the future is fundamental for assessing the challenges facing productive and sustainable agriculture (Ritchie et al., 2019).

Long-term observation data (Kendon et al., 2020) in the UK suggests that the most recent decade (2010–2019) has been, on average, 0.3 °C warmer than the period 1981–2010 and 0.9 °C warmer than 1961–1990. Concurrently, winter precipitation has also increased by 4% and 12%, respectively. Recent climate projections for the UK in the 21st century reported in UKCP18 (https://www.metoffice.gov.uk/research/approach/collaboration/ukcp/download-data) suggest a continued trend of increased likelihood of warmer, wetter winters and hotter, drier summers, along with an increase in the frequency of weather extremes (Chan et al., 2018; Met Office, 2019). Pollution from intensive farming generates off-site environmental damage with resultant costs generated for society, including for example, those for drinking water treatment to remove nutrients and sediment (Eory et al., 2013). Elevated pollution driven by extreme wet-weather increases such negative externalities. Our work aimed to document those externalities for both nitrate and sediment.

February 2020 was the wettest February on record for the UK with the meteorological winter (December, January, February) 2020 ranked as the 5th wettest winter on record since 1862 (e.g.,https://www.metoffice.gov.uk/about-us/press-office/news/weather-and-climate/2020/2020-winter-february-stats). Importantly, England and Wales also experienced a wetter than average October and November 2019 prior to the extreme wet winter. Rainfall rather than snowfall dominates winter precipitation in the UK.

High temporal resolution surface water quality data were collected throughout the extreme wet-weather period (October 2019–March 2020) at a purpose-built farm (North Wyke Farm Platform; NWFP) and landscape scale (Upper River Taw Observatory; UTRO) monitoring platform in SW England, encapsulating both livestock and arable farming systems. The former has multiple hydrologically-isolated field-scale catchments and the latter has nested catchments of varying sizes. Our overarching objectives were: (i) to quantify runoff, water quality responses and environmental damage costs at field and landscape scales during the 2019–2020 extreme wet-weather period, compared to preceding monitored years (2016–2019); (ii) to compare the climatic baseline (1981–2010), extreme wet-weather period (2019–2020) and projected near- (2041–2060) and far- (2071–2090) future climates using conventional rainfall indices, to assess the likelihood of similar wet-weather occurring again, and; (iii) to explore relationships between the conventional rainfall indices and monitored nitrate and sediment responses during the extreme wet-weather period to confirm whether the monitored responses can provide any insight into the externalities that might be expected from agricultural runoff under future climates.

## 2 Materials and methods

### 2.1 Monitoring sites

**Fig. 1:**
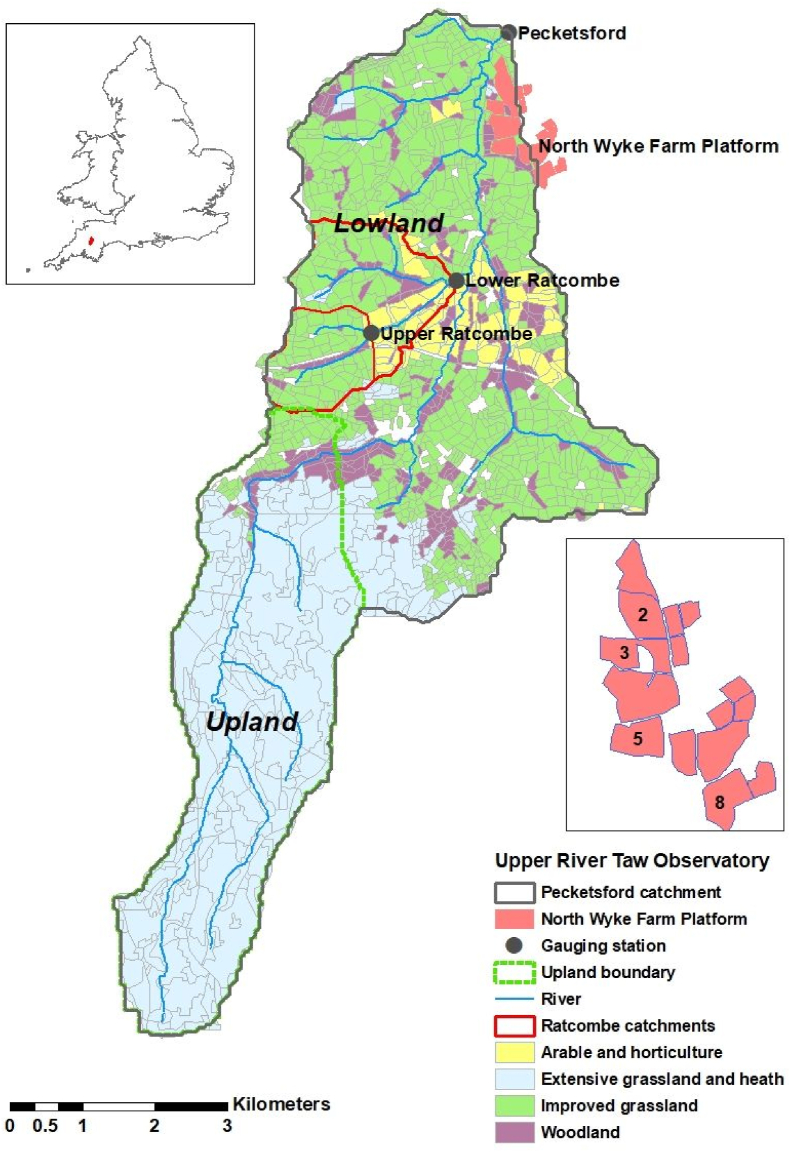
Map showing the study location in SW England, upland and lowland areas, field-scale catchment numbers on the NWFP and hydrological monitoring stations at the outlet of the landscape scale catchments in the URTO.

The field and landscape scale study sites are situated in the upper reaches of the River Taw catchment, south west England (Fig. 1). Long-term (1981–2010) annual average rainfall (Met Office, 2018) is up to 2468 mm in the upland area, compared with 1009 mm at the outlet of the URTO (upstream of 50°46′47.6″N, 3°54′18.3″W). Most of the precipitation falls in the winter and the climate is typical of temperate Atlantic Britain (5–14 °C).

#### 2.1.1 Field-scale sites on the NWFP

The NWFP (50°46′10″N, 3°54′05″W; Fig. 1, photos in supplementary information and http://resources.rothamsted.ac.uk/sites/default/files/groups/North_Wyke_Farm_Platform/FP_UG.Doc_.001_EstabDevelop_ver1.6.pdf) is a UK National Capability where measurements of rainfall, flow and water chemistry at 15-min intervals are undertaken in field-scale (∼7 ha each) hydrologically-isolated catchments using state-of-the-art monitoring infrastructure and sensors (Orr et al., 2016). There is also a weather station managed by the UK Met Office, UK (site name ‘North Wyke’), which has been in operation since 1980. Published data for the four field-scale catchments were downloaded from the NWFP data portal (https://nwfp.rothamsted.ac.uk/) for the period spanning October 2016–March 2020. Field-scale catchments 2,3,5 and 8 were used given their relative high data coverage and contrasting land uses (see land use information in Tables A2-A5).

#### 2.1.2 Landscape scale sites in the URTO

The URTO encompasses two (Upper Ratcombe – 1.7 km^2^ and Lower Ratcombe – 4.4 km^2^) small sub-catchments and the overall outlet (41.4 km^2^) at Pecketsford (Fig. 1 and photo in supplementary information). General topographical and hydrological characteristics are summarised in Table A1. The soils of the lowland portions of the study catchment are poorly draining clay-rich gley soils and typical brown earths, while the soils on the Dartmoor upland at the river source consist of peat and podzols. River hydrology is surface water driven, reflecting the low permeability of the soils, sub-soils, and lithology and, as a result, river discharge responds rapidly to rainfall.

### 2.2 Water quality monitoring data collection and quality control

Field-scale discharges on the NWFP are measured using a combination of H-type flumes [TRACOM Inc., Georgia, USA] and pressure level depth sensors [OTT hydromet, Loveland, CO., USA]. Each field-scale catchment has a flume cabin which houses telemetry devices, pumps, and a by-pass flow cell containing water quality sensors. Multi-parameter sondes [originally YSI 6600V2 and latterly YSI Xylem, Inc Rye Brook, New York, U.S] are used for monitoring turbidity. Suspended solids are determined through the change in mass of a pre-weighed GF/C (Whatman, Buckinghamshire, U.K.) filter paper, with a particle retention size of 1.2 μm, following the vacuum filtration of a known sample volume and subsequent drying at 105 °C {UK Standing Committee of Analysts, 1980 #597}. Ratings of paired sonde readings for turbidity and filtered water sample solids masses are used to convert the former into suspended sediment concentrations. Combined nitrate-N and nitrite-N (NOx-N) are measured by a dedicated, self-cleaning, optical UV absorption sensor [NITRATAX Plus SC, Loveland, Colorado, USA].

At landscape scale in the URTO, river discharge is gauged with streambed mounted sensors within a surveyed channel section. Water velocity is measured using an ultrasound sensor (Mainstream Measurements LTD, U.K.), while water level is measured using a pressure sensor (OTT Hydrometry, U.K.). The combined outputs are sent to a flow transmitter (Mainstream Measurements LTD, U.K.) which using the water level, cross sectional area and water velocity, calculates discharge.

Multi-parameter YSI 6600V2 sondes deployed at the URTO monitoring sites are returned to the laboratory monthly for cleaning and recalibration. The nitrate-N ISE is placed in a 5 mg N l^−1^ solution and the value it measures is recorded, both pre- and post-calibration. The nitrate-N ISE is replaced every 3–4 months, or when performance is unsatisfactory. The deviation of pre- and post- values from the expected standard value (5 mg l^−1^) is used to correct any drifts linearly. Suspended sediment concentrations are determined using the same procedures described above. In stream measurements of flow and water quality are controlled, and data recalled, via Adcon (ADCON, Austria) remote telemetry units using UHF radio every 15-min.

Storm sampling is undertaken at the URTO sites using ISCO 3700 automatic water samplers (Teledyne ISCO, Lincoln, Nebraska, U.S.) and laboratory analyses on these samples are used for developing ratings for converting sonde readings into pollutant concentrations (e.g., Pulley and Collins, 2019). Internal clocks are synchronised prior to sampling and sample intervals are catchment-specific based on the duration and quantity of the rainfall forecast but are always set to coincide with the 15-min sample interval used for the flow and sonde measurements. Samples are stored at 4 °C for analysis. Total nitrogen concentrations are determined through the oxidation of the sample alkaline persulphate in an autoclave at 121 °C to form nitrate. The nitrate is then reduced to nitrite by hydrazine sulphate and total nitrite analysed colourimetrically on an Aquachem 250 analyser through the formation of an azo dye with an absorbance maximum at 540 nm {Hosomi, 1986 #1558}.

### 2.3 Construction of local scale future climate scenarios

Local scale climate scenarios were based on 19 global climate models from the CMIP5 multi-model ensemble (Taylor et al., 2012) used in the IPCC Assessment Report 5 (AR5) (IPCC, 2014). Climate scenarios were generated for the baseline (1981–2010), near-future (2041–2060) and far-future (2071–2090) climates assuming the RCP8.5 representative concentration pathway (Semenov and Stratonovitch, 2015; Table A6). The RCP8.5, business-as-usual or a worst-case emission scenario, combines assumptions about high population and modest technological improvements, leading to high energy demand with the highest greenhouse gas concentration (Riahi et al., 2011). The use of future climate projections from a multi-model ensemble allowed us to estimate uncertainty in our predictions due to uncertainties in climate modelling (Semenov and Stratonovitch, 2010). However, due to the coarse spatial and temporal resolution of GCMs and large uncertainties in the model outputs, it is not appropriate to use daily output directly from GCMs for analysis of extreme weather events (Semenov and Stratonovitch, 2010). Therefore, we used the LARS-WG stochastic weather generator to downscale the climate projections from the GCMs to local scale climate scenarios incorporating changes in both the mean climate and climatic variability derived from the GCMs, by modifying the statistical distributions of the weather variables (Semenov and Stratonovitch, 2015). LARS-WG has been used in many recent European climate change impact and risk assessments (Trnka et al., 2014; Senapati et al., 2020; Senapati and Semenov, 2020), and has been found to perform well in a range of diverse European climates (Semenov, 2008; Semenov et al., 2010; Gitau et al., 2018).

For each selected site, LARS-WG generated 100 years of daily weather for the baseline, near-future and far-future climate scenarios. A large number of years (100) were used to reproduce, accurately, climatic variability and extreme weather events in the observed baseline climate. At Rothamsted Research North Wyke, daily weather observations from 1981 to 2010 were available, which were used by LARS-WG to estimate site parameters of the distributions of climatic variables. These site parameters were used to generate daily baseline weather with the same statistical characteristics as the observed data. For the upland and lowland part of the catchment (cf. Fig. 1), however, observations of daily weather were not available. To obtain site parameters for the baseline climate for these sites, we used the ELPIS dataset (Semenov et al., 2010b). ELPIS is based on the European Crop Growth Monitoring System (CGMS) meteorological dataset and consists of the LARS-WG site parameters for the period 1980–2010 at a spatial resolution of 25 km across Europe. The ELPIS dataset has been validated against daily weather observations obtained independently from the European Climate Assessment & Dataset project (ECA&D) (Semenov et al., 2013). For each of our sites, near-future and far-future local scale climate scenarios were generated by LARS-WG using site parameters for the baseline climate and changes in the distributions of climatic variables derived from individual GCMs for the corresponding near- or far-future periods. LARS-WG 6.0, was used in our study and is available at https://sites.google.com/view/lars-wg/.

### 2.4 Generation and comparison of extreme values for rainfall indices

Selection of daily rainfall-based indicators was based on Dunn et al. (2020). These comprised maximum 1 day rainfall (R1x), number of days with rainfall >1 mm (R1D), >1 mm rainfall amount (R1A), number of days with rainfall >10 mm (R10D), >10 mm rainfall amount (R10A), simple daily intensity index which equals R1A/R1D, maximum consecutive dry days with rainfall <1 mm (CDD1), maximum consecutive dry days with rainfall <10 mm (CDD10), and total rainfall for the study months. The daily threshold value of 10 mm is associated with more erosive rainfall events whereas consecutive wet days can seriously affect ground saturation with concomitant implications for runoff generation, soil erosion and water pollution. The indices were considered appropriate for the study area since it is characterised by seasonally-waterlogged heavy soils meaning that rainfall totals rather than intensities drive hydro-chemical responses. Comparisons of indices were based on 95th percentiles. Two-sample Kolmogorov-Smirnov tests were used to compare the rainfall characteristics between different time periods statistically (alpha of 0.05), namely; baseline (1981–2010), wet weather period (2019–2020), near-future (2041–2060) and far-future (2071–2090). To evaluate the representativeness of the 2019–2020 extreme wet period, in the context of either the baseline or future climates, the closest match of each rainfall index was identified.

### 2.5 Water pollutant loads and associated environmental damage costs

Seven methods (Marsh et al., 2006; see appendix B) were implemented for water pollutant load estimation in recognition that selecting just one algorithm can be arbitrary and to provide a range of estimates for integration with pollutant unit prices (i.e., cost per kg emitted to water). Damage costs were estimated by multiplying pollutant loads with the corresponding unit price (provided by the UK Department for Environment, Food and Rural Affairs). To estimate comparable environmental damage costs for the field and landscape scale catchments, monthly estimates for nitrate and suspended sediment loads were firstly scaled using their respective catchment areas and then the median and Qn (a robust alternative to median absolute deviation; Rousseeuw and Croux, 1993) of the scaled values for each unique combination of site and period were calculated. The use of non-parametric statistics was out of concern for the small size (n = 7) of samples and to reduce the potential effects of outliers. The unit prices were assumed to have a triangular probability distribution with known minimum, typical and maximum values (see Collins and Zhang, 2016 for an explanation of the calculation of the unit prices). Assuming a normal distribution for the water pollutant load estimates, Monte Carlo simulation was implemented with automated routines using @Risk software (version 7.6) to estimate the distributions of environmental damage costs. 5000 Monte Carlo iterations were undertaken using Latin hypercube sampling.

## 3 Results

### 3.1 Runoff responses at field and landscape scales

**Fig. 2:**
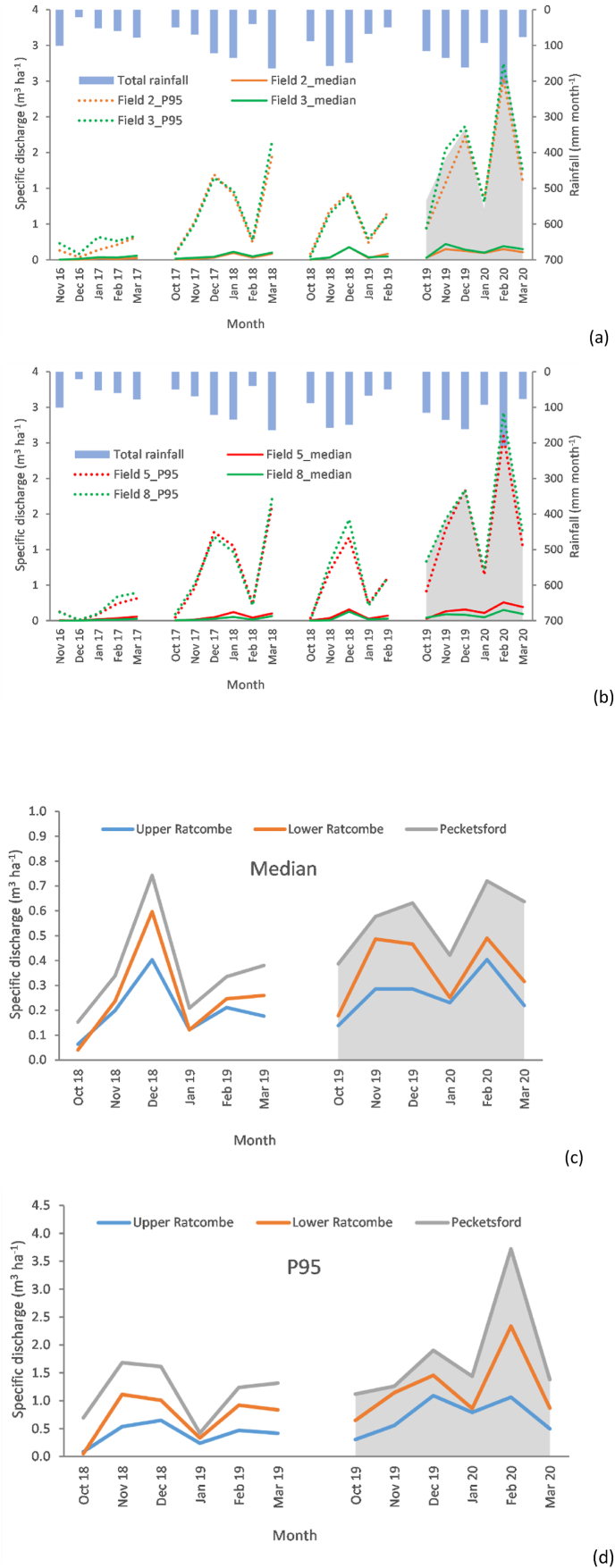
Temporal patterns in monitored flows for 2016–2019: a) monthly summary statistics of flow rates (m^3^ ha^−1^) at field-scale on the NWFP for field catchment 2 and 3; b) monthly summary statistics of flow rates (m^3^ ha^-1^) at field-scale on the NWFP for field catchment 5 and 8; c) monthly median flows (m^3^ ha^−1^) at landscape scale in the URTO, and; d) monthly 95th percentile flows (m^3^ ha^−1^) at landscape scale in the URTO. Field-scale catchment numbers and landscape scale monitoring station names correspond to those in Fig. 1. Grey shaded area depicts the extreme wet weather period in 2019–2020.

Summary statistics for field-scale flow rates for 2016–2020 are tabulated in Table A7A. All field-scale flow regimes spanning October–March 2016–2019 exhibited similar monthly trends (Fig. 2a and b) with significant positive correlations (r > 0.77) between monthly rainfall and average flow (converted to m^3^ ha^−1^ for comparison with landscape values) and monthly rainfall and 95th percentile flow rates. Average monthly flow rates over the 2016–2019 study months were very similar, ranging from 1.0 to 1.1 l s^−1^. In contrast, October–March 2019–2020 was characterised by above average (2016–2019) flow rates for most of the focus months in all four fields. Of note, February 2020 resulted in 3.7- to 5.8-fold increases in average, median and 95th percentile flow rates compared to the corresponding averages for 2016–2019. Observed flow rates at the three monitored catchments in the URTO were scaled by their respective catchment areas (m^3^ ha^−1^; Fig. 2c and d). The temporal patterns at landscape scale were similar to those at field scale, with the extreme wet-weather in 2019–2020 manifested in elevated runoff.

### 3.2 Water pollutant concentrations at field and landscape scales

**Fig. 3:**
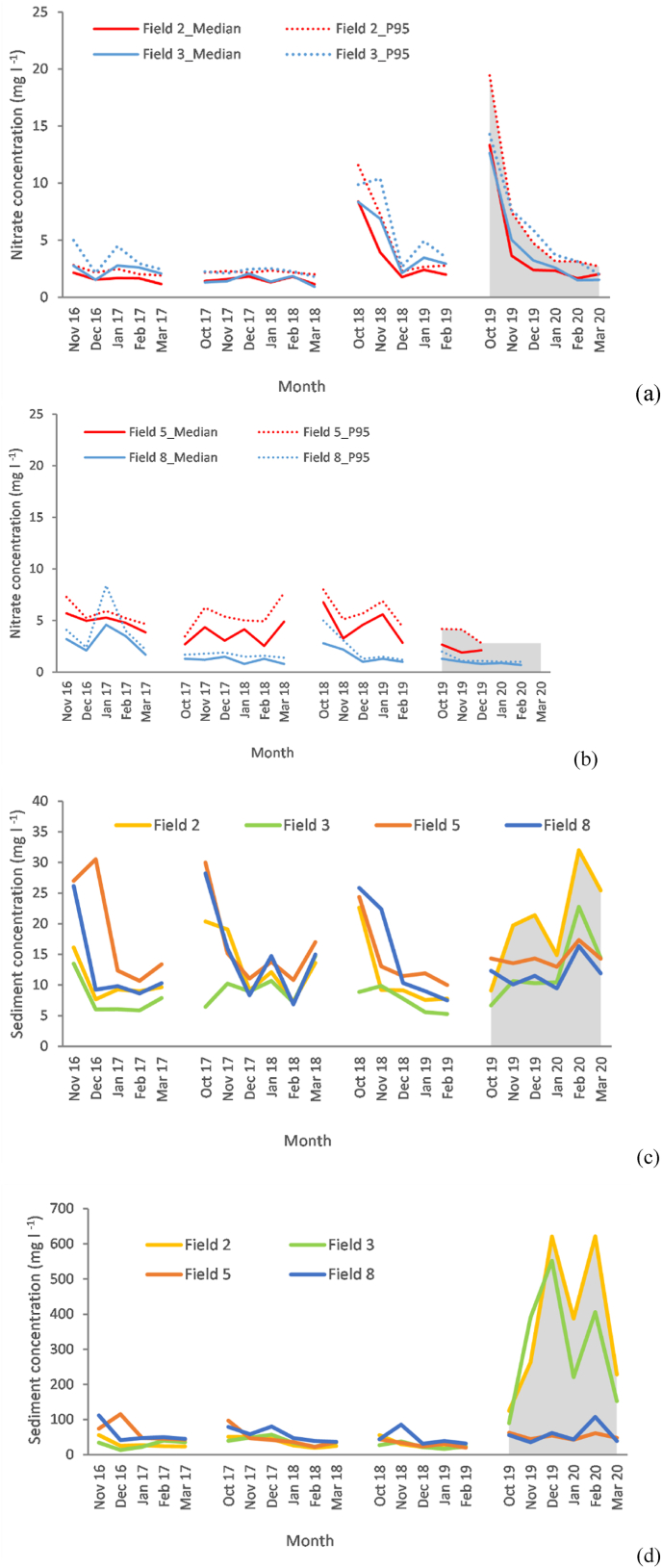
Monthly variations in water pollutant concentrations at field-scale on the NWFP: a) summary statistics of monthly nitrate concentrations for field catchment 2 and 3; summary statistics of monthly nitrate concentrations for field catchment 5 and 8; c) monthly medians of suspended sediment concentrations, and; d) monthly 95th percentiles of suspended sediment concentrations. Field-scale catchment numbers and landscape scale monitoring station names correspond to those in Fig. 1. Grey shaded area depicts the extreme wet weather period in 2019–2020.

Field-scale average monthly nitrate concentrations (Table A7B) were <3 mg N l^−1^ for most months (Fig. 3a). For 2016–2019, average concentrations ranged between 2.0 and 4.2 mg N l^−1^ (coefficients of variance 25–80%). The similarity between the mean and median values and the subdued increase from median values to the corresponding 95th percentiles across all fields suggests a steady and gradual delivery process, which is typical of the subsurface pathway. During 2019–2020, higher nitrate concentrations were recorded in fields 2 and 3 in early winter where long-term improved grassland was ploughed and sown into winter wheat. For a limited time, these concentrations even exceeded the recommended threshold value (11.3 mg N l^−1^) stipulated in the EC Nitrate Directive (https://ec.europa.eu/environment/water/water-nitrates/index_en.html). The wet-weather in February 2020 resulted in no significant impacts on median and 95th percentile nitrate concentrations for fields 5 or 8, meaning that the land conversion from grass to cereals in fields 2 and 3 resulted in a more pronounced response over the extreme wet period in 2019–2020 (Fig. 3a). The average concentrations in 2019–2020 were ∼4 mg N l^−1^ for the fields converted from grass to arable compared to 1–2 mg N l^−1^ for the fields still in grass. In contrast, suspended sediment concentrations (Table A7C) demonstrated more variation (Fig. 3b and c). During 2016–2019, average concentrations ranged between 12.5 and 21.2 mg l^−1^ (coefficients of variance 30–40%). The substantial differences between the median and 95th percentile values highlight the effects of individual short-interval storm events. First flushes were evident in the early months at all fields during which average suspended sediment concentrations exceeded 20 mg l^−1^.

During 2019–2020, the magnitudes and temporal patterns of the suspended sediment concentrations (Table A7C) changed significantly in fields 2 and 3 where land use conversion to cereal production occurred (Fig. 3c and d). Here, median concentrations were 76.2 mg l^−1^ and 65.1 mg l^−1^, respectively. The highest monthly average concentration of 133.2 mg l^−1^ was recorded in field 2 in February 2020. The most significant change concerned the estimated 95th percentiles which exceeded 150 mg l^−1^ continuously from November 2019. Peaks of >600 mg l^−1^ were recorded in both December 2019 and February 2020 in fields 2 and 3 converted to arable production. There was no significant increase in suspended sediment concentrations over any of the winters in fields 5 and 8 which remained as permanent grassland.

**Fig. 4:**
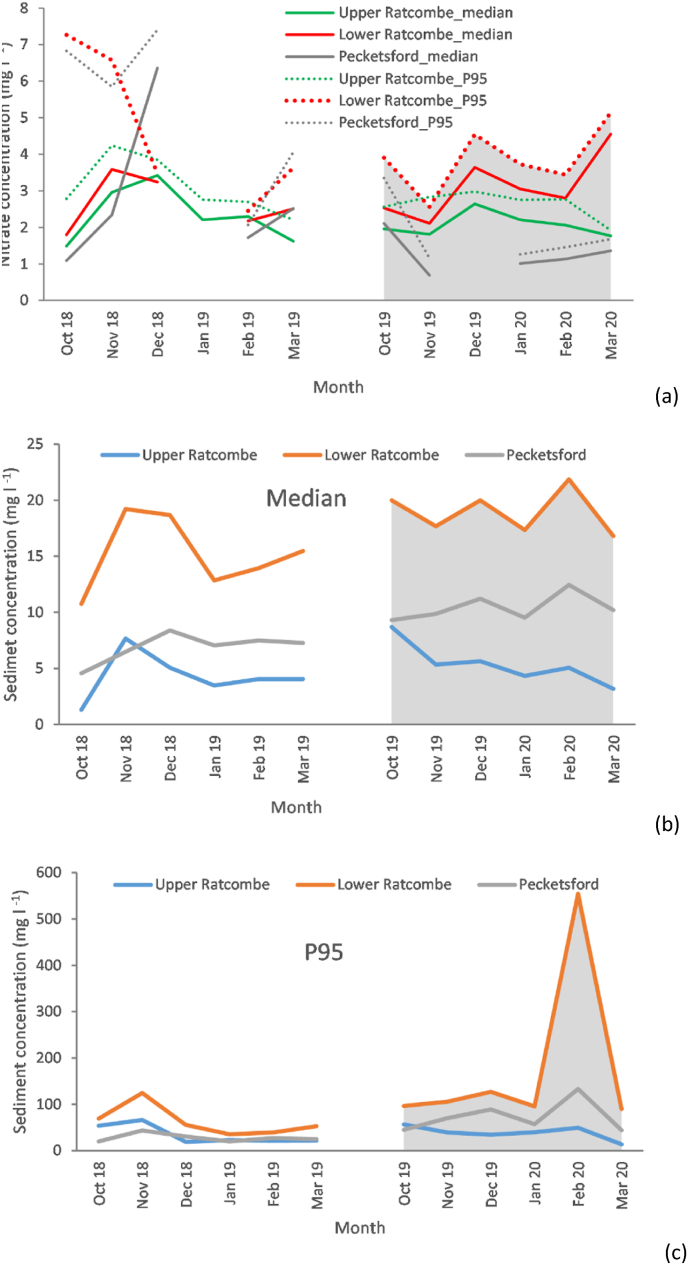
Monthly variations in water pollutant concentrations at landscape scale in the URTO: a) monthly summary statistics of nitrate concentrations; b) monthly median suspended sediment concentrations; and c) monthly 95th percentiles of suspended sediment concentrations. Landscape scale monitoring station names correspond to those in Fig. 1. Grey shaded area depicts the extreme wet weather period in 2019–2020.

Fig. 4 presents nitrate and suspended sediment concentrations in the URTO. Median nitrate concentrations were still very low, rarely exceeding 5 mg l^−1^. During the wetter 2019–2020 period, the median nitrate concentrations at Lower Ratcombe were slightly higher than those at Upper Ratcombe. Differences between the two sites were most pronounced in March 2020 when the estimated median monthly nitrate concentrations were 4.6 mg l^−1^ and 1.7 mg l^−1^, respectively. More limited data from Pecketsford suggest that the nitrate concentrations further downstream were even lower. The small increase in 95th percentile concentrations above the corresponding median values across the monitoring period (2018–2020) was similar to the trend observed at field scale (Fig. 4a). Monthly median values varied between 1.3 and 8.7 mg l^−1^, 10.8–21.9 mg l^−1^ and 4.6-12.5 mg l^−1^ at Upper Ratcombe, Lower Ratcombe and Pecketsford, respectively. The much higher median suspended sediment concentrations at Lower Ratcombe reflect an increased proportion of arable land compared to the Upper Ratcombe catchment (Table 1). Subdued inter-month variations were observed at both Upper Ratcombe and Pecketsford, but sharp variations were recorded at Lower Ratcombe. The Upper Ratcombe monitoring station exhibited an insignificant change in monthly median suspended sediment concentrations even in the very wet February 2020, whereas both Lower Ratcombe and Pecketsford exhibited substantial elevations (Fig. 4b). Heavy rainfall in February 2020 elevated the 95th percentiles of suspended sediment concentrations to 49.3 mg l^−1^ at Upper Ratcombe, 554 mg l^−1^ at Lower Ratcombe and 133.3 mg l^−1^ at Pecketsford (Fig. 4c). Average sediment concentrations in 2019–2020 exhibited respective increases of 13%, 184% and 164% relative to the estimates for 2018–2019.

**Table 1:** Estimated cumulative environmental damage costs at field-scale on the NWFP.

Pollutant	Field	Water	Average	5th percentile	95th percentile	Standard deviation
		year	(£ ha^−1^)	(£ ha^−1^)	(£ ha^−1^)	(£ ha^−1^)
Nitrate	Field 2	2016	0.5	0.4	0.7	0.1
		2017	0.8	0.5	1.1	0.2
		2018	1.9	1.3	2.4	0.3
		2019	3.4	2.4	4.5	0.6
	Field 3	2016	0.9	0.5	1.2	0.2
		2017	1	0.7	1.3	0.2
		2018	2.7	1.9	3.5	0.5
		2019	5.7	3.8	7.8	1.2
	Field 5	2016	1.1	0.5	1.9	0.4
		2017	2	1.4	2.6	0.4
		2018	2.9	2.1	3.9	0.5
		2019	2.4	1.7	3.1	0.4
	Field 8	2016	0.7	0.4	1.2	0.2
		2017	0.5	0.3	0.7	0.1
		2018	0.9	0.6	1.2	0.2
		2019	1.3	0.9	1.6	0.2
Sediment	Field 2	2016	1.1	1	1.2	0.1
		2017	4.7	4.2	5.2	0.3
		2018	2.5	2.3	2.8	0.1
		2019	108	95.6	120.9	7.7
	Field 3	2016	1.1	0.9	1.2	0.1
		2017	6.9	6.1	7.6	0.4
		2018	3	2.7	3.3	0.2
		2019	115.5	103.5	128	7.4
	Field 5	2016	1.7	1.1	2.2	0.3
		2017	6	5.3	6.8	0.4
		2018	3.6	3.3	4	0.2
		2019	13	11.7	14.4	0.8
	Field 8	2016	1.7	1.3	2.2	0.3
		2017	7.1	6.2	8	0.5
		2018	5.5	4.9	6	0.4
		2019	15.7	12.9	18.7	1.8

### 3.3 Water pollutant loads at field and landscape scales

**Fig. 5:**
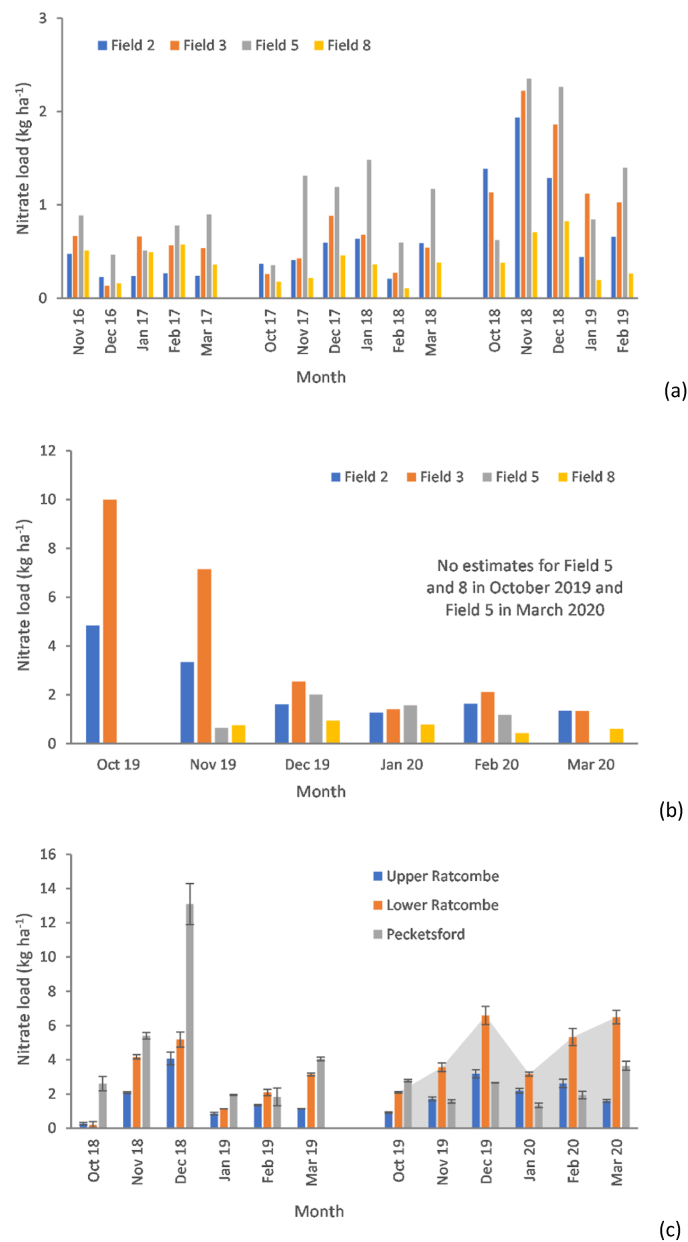
Estimated monthly nitrate loads: a) monthly nitrate loads at field-scale on the NWFP for 2016–2019; b) monthly loads at field-scale on the NWFP for 2019–2020, and; c) monthly loads at landscape scale in the URTO. Field numbers and landscape scale monitoring station names correspond to those in Fig. 1. Data for October 2016 in Fig. 5a are missing.

Nitrate loads are summarised in Fig. 5. Grassland field-scale average nitrate losses (Fig. 5a and b) increased from 0.39-1.07 kg ha^−1^ (2016–2019) to 0.70–1.35 kg ha^−1^ (2019–2020), whereas losses from long-term grassland grazed by beef and sheep ploughed up for winter cereal cropping, increased from 0.63-0.83 kg ha^−1^ to 2.34–4.09 kg ha^−1^. Nitrate losses at landscape scale (Fig. 5c) increased during the 2019–2020 extreme wet-weather period to between 2.04 and 4.54 kg ha^−1^. During 2017–2018, the same losses were estimated to be 1.63–4.83 kg ha^−1^. The field-scale nitrate load estimates clearly illustrate the combined effects of extreme wet-weather and land use conversion from grass to arable in elevating emissions to water. Appendix B summarises all nitrate load estimates.

**Fig. 6:**
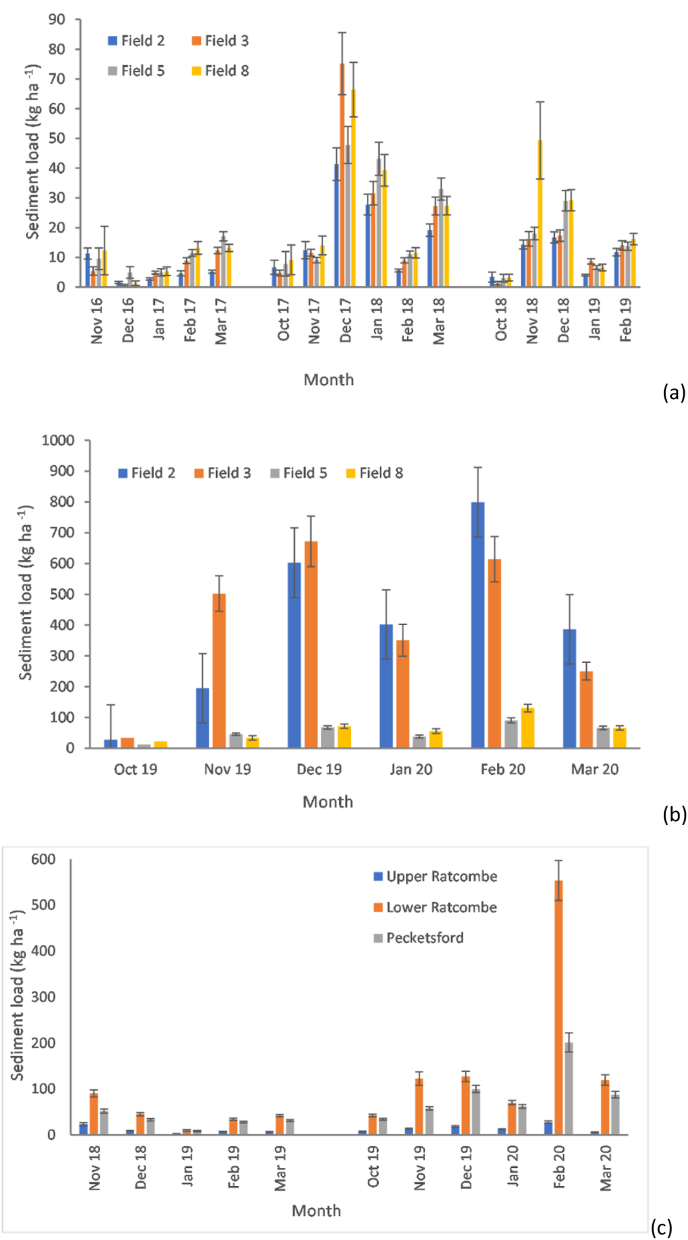
Estimated monthly suspended sediment loads at field-scale on the NWFP: a) monthly suspended sediment loads for 2016–2019; b) monthly suspended sediment loads for 2019–2020, and; c) monthly suspended sediment loads at landscape scale in the URTO. Field numbers and landscape scale monitoring station names correspond to those in Fig. 1. Data records for October 2016 on the NWFP and October 2018 on the URTO were too sparse to generate estimates for use in the above plots.

Suspended sediment loads (Fig. 6a) during 2016–2019 for fields 2 and 3 ranged between 29 and 138 kg ha^−1^ compared to 52–162 kg ha^−1^ for fields 5 and 8. For the months in 2019–2020 (Fig. 6b), fields 5 and 8 exhibited a three-fold increase (92 kg ha^−1^ to 281 kg ha^−1^ for the former and 116 kg ha^−1^ to 333 kg ha^−1^ for the latter) in loads compared with the overall average for 2016–2019. Comparing 2016–2019 and 2019–2020, the corresponding total loads increased from 63 kg ha^−1^ to 2146 kg ha^−1^ in field 2 and from 80 kg ha^−1^ to 2124 kg ha^−1^ in field 3. The field-scale suspended sediment loads underscore the combined effects of extreme wet-weather and land use conversion from long-term grass to arable in elevating emissions to the aquatic environment. Appendix B summarises all sediment load estimates.

Fig. 6c compares landscape scale suspended sediment loads in the URTO for 2018–2019 and 2019–2020. The most striking feature is the substantial increase in exported load at Lower Ratcombe in February 2020 when the estimated monthly load exceeded 550 kg ha^−1^. The elevated sediment export was, however, lower than the corresponding estimated elevated loads for fields 2 (799 kg ha^−1^) and 3 (614 kg ha^−1^) on the NWFP which had undergone conversion to arable production. The landscape scale monthly suspended sediment loads ranged between 42 kg ha^−1^ and 553 kg ha^−1^ at Lower Ratcombe and 9–201 kg ha^−1^ at Pecketsford. Compared with 2018, the overall average suspended sediment load at Upper Ratcombe only increased by 45% but by 288% and 196% during the extreme wet-weather in 2019–2020. Appendix B summarises all landscape load estimates.

### 3.4 Environmental damage costs due to water pollution at field and landscape scales

Water pollutant emissions affect the provision of valuable ecosystem services and these impacts can be assessed using environmental damage costs. Table 1 presents the estimated damage costs for the field scale water pollutant emissions on the NWFP and Table 2 those at landscape scale in the URTO. For field-scale nitrate emissions, the estimated average damage costs were £3 ha^−1^ for the period 2016–2019. Three fields generated slightly elevated damage costs in the wetter period in 2019–2020, with those costs for fields 2 and 3 increasing to £3.4 ha^-1^ and £5.7 ha^-1^, respectively. At landscape scale in the URTO, the highest damage costs were estimated at Pecketsford in the 2018–2019 winter at £12.4 ha^-1^. The damage costs remained almost unchanged at Upper Ratcombe (£4.2 ha^-1^ for 2016–2019 and £5.3 ha^-1^ in 2019–2020), compared with a 71% increase at Lower Ratcombe (£6.9 ha^-1^ to £11.7 ha^-1^).

**Table 2:** Estimated cumulative environmental damage costs at landscape scale in the URTO.

Pollutant	Catchment	Water year	Average (£ ha^−1^)	5th percentile (£ ha^−1^)	95th percentile (£ ha^−1^)	Standard deviation (£ ha^−1^)
Nitrate	Upper Ratcombe	2018	4.2	2.9	5.6	0.8
		2019	5.3	3.6	7.0	1.0
	Lower Ratcombe	2018	6.9	4.7	9.3	1.4
		2019	11.7	8.2	15.3	2.1
	Pecketsford	2018	12.4	8.1	17.3	2.8
		2019	6.0	4.1	8.0	1.2
Sediment	Upper Ratcombe	2018	2.7	1.9	3.6	0.5
		2019	4.7	4.0	5.3	0.4
	Lower Ratcombe	2018	12.1	10.9	13.2	0.7
		2019	56.0	49.7	62.2	3.8
	Pecketsford	2018	9.0	8.2	9.9	0.5
		2019	29.3	25.7	33.0	2.2

For field-scale suspended sediment emissions on the NWFP, corresponding environmental damage costs were generally less than £8 ha^−1^ during 2016–2019. During the wetter period spanning 2019–2020, however, the costs increased by 3-fold for fields 5 and 8 but by more than 30-fold to ∼£100 ha^−1^ for fields 2 and 3 which had been converted to arable production. For the three catchments in the URTO, the environmental damage costs followed the following ranking in both 2018–2019 and 2019–2020: Lower Ratcombe > Pecketsford > Upper Ratcombe; but there were significant differences in their relative increases during 2019–2020 compared with 2018–2019. Here, the relative increases were 364% (from £12.1 ha^-1^ to £56.0 ha^-1^) at Lower Ratcombe, 224% (from £9.0 ha^-1^ to £29.3 ha^-1^) at Pecketsford and 74% (from £2.7 ha^-1^ to £4.7 ha^-1^) at Upper Ratcombe.

### 3.5 Comparison of current extreme wet weather with baseline and future climates using rainfall indices and relationships with pollutant losses

**Fig. 7:**
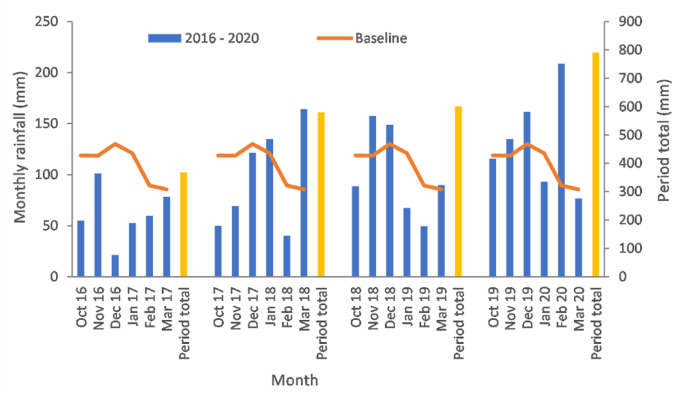
Comparison of October–March rainfall for 2016–2020 with the climatic baseline (1981–2010).

For rainfall-driven diffuse water quality responses, the first flush of potential pollutants associated with the soil ‘wetting up’ in the UK, typically occurs in mid to late autumn. Accordingly, our comparison of rainfall records for different time periods focussed on the months October–March inclusive, rather than only the meteorological (December–February) winter. Climatic baseline (1981–2010) data for the study location suggest an average rainfall total of ∼633 mm for these six months (Fig. 7). October–March 2016–2017 was very dry with only ∼56% of the climatic baseline rainfall, whereas 2017–2018 and 2018–2019 experienced near baseline totals. In contrast, 2019–2020 was much wetter with nearly 20% more rainfall than the climatic baseline. Whilst November and December 2019 experienced >15% more rainfall than the climatic baseline, ∼209 mm fell in February 2020 (>133% more than the climatic baseline; the third highest monthly rainfall on record since 1982). On the basis of total rainfall, >1 mm rain days, >10 mm rain days and maximum 5-days rainfall, the return period of the 2019–2020 six month wet weather period is less than 1 in 80 years.

Looking ahead to near- (2041–2060) and far- (2071–2090) climatic future scenarios, analysis of the same rainfall indices (Table 3) suggests small but uncertain changes in both the upland and lowland parts of the study catchment shown in Fig. 1. Most indices show fewer than half (<10 out of 19) of the ensemble members returning either significant positive or negative changes relative to the 1981–2010 climatic baseline. Relatively speaking, more consensus is projected for the upland part of the study catchment in the far future wherein ≥10 ensemble members predict an increase in >1 mm rainfall, >10 mm rainfall, simple daily density index, and maximum 5-day rainfall. Only ≤3 ensemble members predict a decrease. For the lowland part of the study catchment, the equivalent consensus suggests only a decrease in maximum 5-day rainfall and total rainfall in the near future.

**Table 3:** Comparison of rainfall indices for near- (2041–2060) and far- (2071–2090) climatic futures with the climatic baseline (1981–2010).

Rainfall indices	Time	Direction of	Upland*	Minimum	Median	Maximum	Lowland*	Minimum	Median	Maximum
			Ensemble				Ensemble			
	period	Change	count	change	change	change	count	change	change	change
>1 mm rain (mm)	2060	Increase	5	57.6	75.3	112.0	5	62.9	76.5	120.0
	2060	Decrease	3	29.1	39.0	51.4	3	31.6	41.6	48.7
	2090	Increase	10	35.8	69.7	177.8	9	54.5	82.8	171.5
	2090	Decrease	3	16.7	56.2	63.4	2	49.3	52.0	54.8
SDII (mm/day)	2060	Increase	5	0.4	0.6	1.1	5	0.4	0.6	1.0
	2060	Decrease	3	0.3	0.3	0.3	3	0.2	0.3	0.4
	2090	Increase	13	0.3	0.5	1.7	8	0.3	0.6	1.6
	2090	Decrease	2	0.4	0.5	0.5	2	0.3	0.4	0.5
>10 mm days (days)	2060	Increase	5	2.7	3.2	4.9	5	2.6	3.2	4.8
	2060	Decrease	5	2.7	3.2	4.9	3	1.5	2.2	2.3
	2090	Increase	8	1.5	3.9	6.9	8	2.0	4.1	7.2
	2090	Decrease	2	2.6	2.9	3.2	2	2.5	2.6	2.7
>10 mm rain (mm)	2060	Increase	6	40.8	78.8	123.7	5	57.2	68.7	115.5
	2060	Decrease	3	24.8	39.2	49.5	3	30.4	44.5	54.1
	2090	Increase	13	32.9	62.1	195.9	9	38.3	77.5	178.5
	2090	Decrease	2	54.7	60.4	66.1	2	50.3	53.2	56.1
Max 1 day (mm)	2060	Increase	5	3.7	4.7	8.0	2	1.7	4.6	7.4
	2060	Decrease	2	1.7	2.5	3.2	6	2.2	3.7	6.1
	2090	Increase	6	3.0	8.4	14.6	3	2.2	7.1	7.2
	2090	Decrease	2	4.0	4.8	5.6	6	2.8	3.6	6.6
CDD1 (days)	2060	Increase	6	1.0	1.5	1.6	3	0.9	1.4	1.7
	2060	Decrease								
	2090	Increase	4	0.9	1.2	1.8	7	0.6	1.2	1.9
	2090	Decrease								
Max 5 days (mm)	2060	Increase	8	3.4	11.3	17.0	4	2.7	8.8	14.8
	2060	Decrease					4	2.6	5.7	8.5
	2090	Increase	11	6.1	9.7	28.8	9	3.3	5.1	20.5
	2090	Decrease					3	5.0	6.4	7.4
Total rainfall (mm)	2060	Increase	5	56.0	73.7	111.2	2	64.4	70.9	77.5
	2060	Decrease	3	29.6	38.8	51.2	8	30.0	46.7	89.1
	2090	Increase	10	35.3	68.7	176.5	5	40.2	68.5	128.2
	2090	Decrease	3	17.7	55.0	62.9	5	51.0	59.2	95.4

*Upland and lowland refer to the areas shown in Fig. 1.

**Fig. 8:**
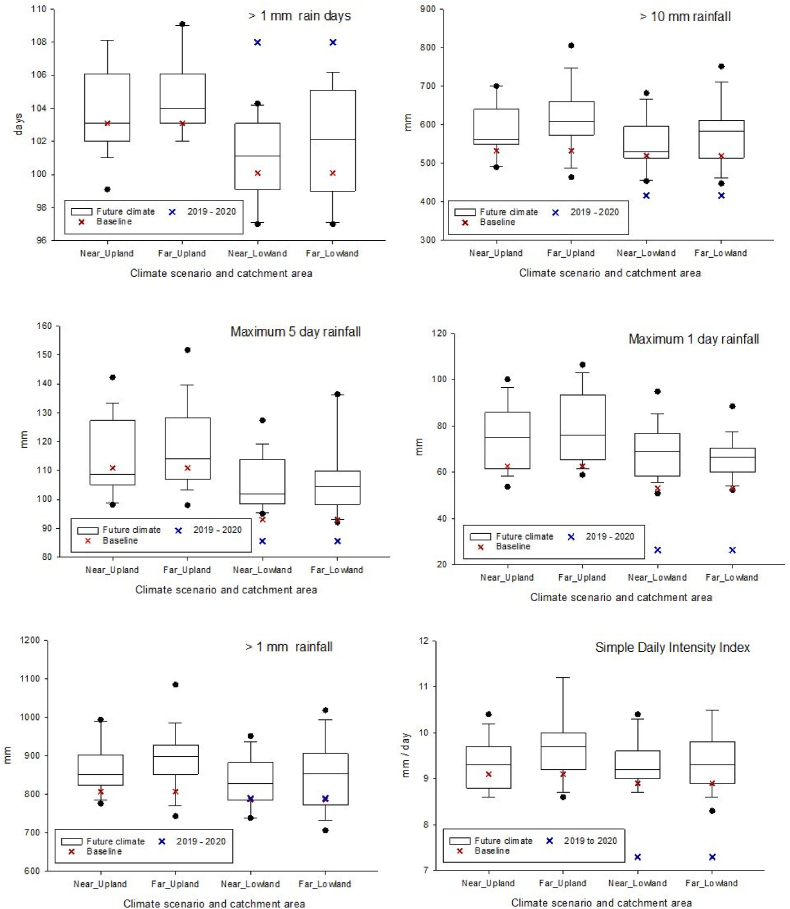
Comparison of the 95th percentiles of different rainfall indices for the climatic baseline (1981–2010), extreme wet weather period (2019–2020) and near- (2041–2060) and far- (2071–2090) future climate scenarios. Box plots are constructed out of 19 predictions for climate scenarios derived from 19 individual GCMs from the CMIP5 ensemble. Box boundaries indicate the 25th and 75th percentiles, the line within the box marks the median, whiskers below and above the box indicate the 10th and 90th percentiles and dots correspond to outliers.

**Fig. 9:**
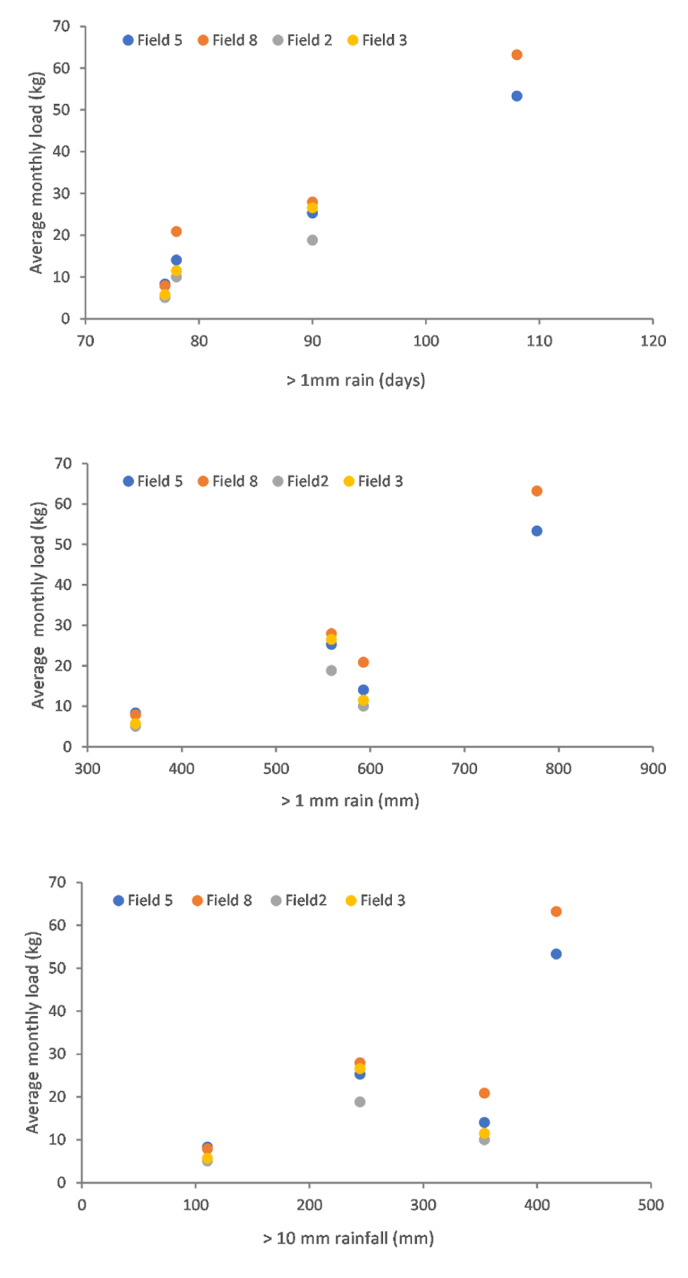
Average monthly suspended sediment loads (2016-19 for field catchments 2 and 3; 2016-2020 for field catchments 5 and 8) at field-scale on the NWFP plotted against the rainfall indices.

Fig. 8 compares the 95th percentiles of the different rainfall indices for the climatic baseline (1981–2010), extreme wet weather period (2019–2020) and near- (2041–2060) and far- (2071–2090) future climates. October 2019–March 2020 was most characteristic of predicted future climates with respect to >1 mm rainfall. The same six months were more extreme than future climates on the basis of >1 mm rain days, but less extreme on the basis of the remaining indices (Fig. 8 and Figure A1). Plots of field-scale nitrate loads on the NWFP against the rainfall indices (Figure A2) did not reveal strong relationships. In contrasts, the same plots for field-scale suspended sediment loads (Fig. 9) suggested stronger relationships, especially in the case of >1 mm rainfall; the rainfall index with the greatest similarity between October 2019–March 2020 and future climates.

## 4 Discussion

Evidence for many parts of the UK suggests that the frequency, duration and event totals of rainfall over winter months have increased (Riahi et al., 2011; Thompson et al., 2017). The importance of extreme rainfall event totals for soil erosion has been underscored by previous work (Boardman, 2015). These changes in autumn and winter rainfall are, in turn, elevating runoff and the water pollution externalities arising from contemporary intensive grassland and cereal agroecosystems, since current on-farm mitigation strategies, including those subsidised by agri-environment schemes, are delivering limited efficacy (Collins and Zhang, 2016; Ockenden et al., 2017; Collins et al., 2021).

October 2019–March 2020 experienced higher than average rainfall totals compared with the 1981–2010 climatic baseline, resulting in elevated water pollution externalities from both grass, but in particular, arable land, at both field and landscape scales. The forecast impacts of climate change on hydrological systems is less clear at local scale in the UK, where weather patterns are strongly influenced by the North Atlantic Oscillation. Accordingly, Global Climate Model (GCM) outputs need to be downscaled to reflect the local interplay between climate and weather processes (Watts et al., 2015). Although the wet-weather period in 2019–2020 is only representative of projected rainfall extremes for near- (2041–2060) and far- (2071–2090) future climates in terms of >1 mm rainfall, we found a strong correlation between this particular rainfall index and monitored suspended sediment loads. Projections of changing rainfall patterns remain very uncertain (IPCC, 2014), but, regardless, consistently predict temporally uneven regimes with increasing dominance of few large events (Pendergrass and Knutti, 2018) and such events are important for soil erosion and sediment delivery in the study area (Upadhayay et al., 2021). High runoff events and concomitant diffuse water pollution are therefore likely to be exacerbated in future climates and the recent wet-weather in 2019–2020 and monitored water quality responses potentially provide some insight into the potential magnitude of suspended sediment losses from intensively managed grass and arable land under future rainfall regimes. Here, it also important to acknowledge that fine-grained sediment exerts a key control on the redistribution and fate of other aquatic pollutants including phosphorus, heavy or trace metals, some pesticides and additional inorganic or organic substances (Meharg et al., 1999; Warren et al., 2003; Liao et al., 2020).

The magnitude of water pollution externalities and associated environmental damage costs arising from extreme wet periods clearly depend on land cover. Although, under the UK Climate Projections medium emissions scenario, arable farming is predicted to advance westwards (Fezzi and Bateman, 2015; Ritchie et al., 2019), replacing the current extensive long-term grassland, the monitored water quality responses at field scale on the NWFP and landscape scale in the URTO encompass both grass and, importantly, grass converted to arable land. The responses of grass and arable land to the extreme wet-weather in 2019–2020, compared to the preceding years (2016–2019) are therefore indicative of the potential consequences of projected land use change under future climates. Land cover controls the complex interplay between pollutant source availability and hydrologic connectivity (McMillan et al., 2018). Deployment of high frequency sensors *in situ* and across scales plays a critical role in the continuous monitoring of water quality responses to extreme weather periods. Such monitoring can be used to provide improved mechanistic understanding of water quality responses which, in turn, can help target remedial actions (Kaushal et al., 2018).

Cultivation and exposure of bare soils during the high-risk window of autumn and winter will occur annually on the land used for winter cereal production. In contrast, scheduled ploughing and reseeding of the grassland will only occur every few years. Production of cereals on the soils present at the study site is therefore repeatedly higher risk with regards elevated water pollution and environmental damage costs. Assuming a typical farm size of 103 ha in the study location, the annual gross margin is typically £623 ha^−1^, compared with environmental damage costs of ∼£124 ha^−1^ (combining nitrate and sediment losses and unit prices for damage costs) during extreme wet-weather. However, the challenge, is that even uptake of all available water pollution mitigation measures recommended by policy and estimated to cost ∼£210 ha^−1^ annually, would only provide technically feasible reductions in sediment and nitrate losses to water of ∼22% and ∼28% under typical long-term average climatic conditions (Zhang et al., 2017) and most likely lower reductions in extreme wet-weather. Long-term, it is therefore not recommended to produce winter cereals at the study site in the context of the shift in the UK to increasing public goods and services from agriculture and the insufficient efficacy of current preferred on-farm mitigation measures for controlling pollutant losses to water.

Whilst we focussed on the implications of present day severe wet-weather for diffuse water pollution from agriculture, there remains a concomitant need for multiple stakeholders including farmers, farm advisors, water companies and environmental agencies to plan for so-called ‘compound events’ wherein severe wet and dry periods occur back to back. Such weather patterns have the potential to result in even more disproportionately severe impacts on the externalities arising from agroecosystems (Johnstone et al., 2016; Dodd et al., 2020).

## 5 Conclusion

Extreme wet-weather increases the externalities of contemporary farming on freshwater environments. Prolonged wet periods have increased in frequency relative to the UK climatic baseline and our work reveals a correlation between the extremes of >1 mm rainfall and increased suspended sediment loss, which, in turn, increases environmental damage costs. On the basis of our findings herein, we argue that current sediment loss in extreme wet-weather periods in our study area provides some insight for the likely magnitude of corresponding future externalities, pointing to the need for improved management strategies for increasing the resilience of agroecosystems to the impact of extreme wet-weather on soil erosion and sediment loss.

## CRediT authorship contribution statement

**Y. Zhang:** Methodology, Validation, Writing – original draft, Writing – review & editing. **S.J. Granger:** Investigation, Data curation. **M.A. Semenov:** Investigation, Data curation. **H.R. Upadhayay:** Data curation. **A.L. Collins:** Funding acquisition, Project administration, Supervision, Methodology, Writing – original draft, Writing – review & editing.

## Declaration of competing interest

The authors declare that they have no known competing financial interests or personal relationships that could have appeared to influence the work reported in this paper.
