# Supplementary material for: Diffuse water pollution during recent extreme wet-weather in the UK: Environmental damage costs and insight into the future?
Source: J Clean Prod. 2022 Mar 1;338:130633. doi: 10.1016/j.jclepro.2022.130633 (PMC8872830; doi:10.1016/j.jclepro.2022.130633)
Supplement: Multimedia component 1 [file mmc1.docx]

**Diffuse water pollution during recent extreme wet-weather in the UK: environmental damage costs and insight into the future?**

Zhang, Y.^1*^, Granger, S.G. ^1^, Semenov, M.A.^2^, Upadhayay, H.R. ^1^, Collins, A.L. ^1^

*^1^Sustainable Agriculture Sciences, Rothamsted Research, North Wyke, Okehampton EX20 2SB, UK*

*yusheng.zhang@rothamsted.ac.uk;* [*steve.granger@rothamsted.ac.uk*](mailto:steve.granger@rothamsted.ac.uk)*;* [*hari-upadhayay@rothamsted.ac.uk*](mailto:hari-upadhayay@rothamsted.ac.uk)*;* [*adrian.collins@rothamsted.ac.uk*](mailto:adrian.collins@rothamsted.ac.uk)

*^2^Plant Sciences Department, Rothamsted Research, West Common, Harpenden AL5 2JQ, UK*

*mikhail.semenov@rothamsted.ac.uk*

*corresponding author

**Appendix A**

Table A1: Catchment parameter values (FEH codes capitalised in brackets) and land use (% of area) for the URTO.

| **Catchment descriptor** | **Upper Ratcombe** | **Lower Ratcombe** | **Pecketsford** |
| --- | --- | --- | --- |
| Mean catchment altitude (ALTBAR; m a.s.l.) | 233 | 213 | 297 |
| Dominant aspect of catchment slopes (ASPBAR; degree) | 53 | 76 | 25 |
| Invariability of the aspect of catchment slopes (ASPVAR; ratio) | 0.42 | 0.38 | 0.27 |
| Base flow index using the Hydrology of Soil Types (BFIHOST; ratio) | 0.482 | 0.43 | 0.45 |
| Mean drainage path slope (DPSBAR; m km^-1^) | 109.3 | 88.7 | 109.8 |
| Flood attenuation by reservoirs and lakes (FARL) | 1.000 | 1.000 | 0.992 |
| Fraction of catchment inundated by 100-year flood (FPEXT) | 0.0043 | 0.0199 | 0.047 |
| Longest drainage pathway (LDP; km) | 2.07 | 3.63 | 18.71 |
| Proportion of time soils are wet (PROPWET) | 0.46 | 0.46 | 0.46 |
| Standard percentage runoff using Hydrology of Soil Types (SPRHOST; %) | 35.62 | 38.18 | 35.11 |
| Land use* |  |  |  |
| Upland (%) | na | Na | 38.3 |
| Total area (km^2^) | 1.7 | 4.4 | 41.4 |
| Moorland (%) | na | Na | 38.3 |
| Permanent Grassland (%) | 86.7 | 70.5 | 43.5 |
| Arable (%) | 1.5 | 17.5 | 6.5 |
| Woodland (%) | 7.4 | 10.1 | 9.5 |
| Freshwater (%) | na | Na | 0.1 |
| Urbanised (%) | 4.4 | 1.8 | 2.2 |

*Data generated from UKCEH landcover ArcGIS layer, visually corrected. Moorland combines acid grassland, bog, and dwarf shrub heath categories. Permanent grassland combines both improved grassland, and rough low-productivity grassland. Woodland combines broad leaved, mixed and yew woodland, and coniferous woodland. Urbanised represents built-up areas and gardens, farmyard units, and significant road surfaces.

FEH - Flood Estimation Handbook (FEH; Institute of Hydrology, 1999)

Table A2: Land use and management history for field-scale catchment 2 on the NWFP.

| **Event Date** | **Land cover** | **Field Operation** | **Application** | **Total application** | **Units** |
| --- | --- | --- | --- | --- | --- |
| 05/04/2016 | Grass | Spread Fertiliser | Nitram | 690 | kg |
| 12/05/2016 | Grass | Spread Fertiliser | Nitram | 750 | kg |
| 14/06/2016 | Grass | Spread Fertiliser | Nitram | 731 | kg |
| 19/07/2016 | Grass | Spread Fertiliser | Nitram | 740 | kg |
| 31/10/2016 | Grass | Lime | Calcifert lime | 4039 | kg |
| 05/04/2017 | Grass | Spread Fertiliser | 22-4-14-7 | 2420 | kg |
| 18/04/2017 | Grass | Spread Fertiliser | 0-20-30 | 657 | kg |
| 20/04/2017 | Grass | Apply Pesticide | Spray Herbicides | 13.3 | l |
| 21/04/2017 | Grass | Spread Fertiliser | Nitram | 765 | kg |
| 08/06/2017 | Grass | Spread Organic Manure | Organic FYM | 50.6 | mt |
| 12/06/2017 | Grass | Spread Organic Manure | Organic FYM | 41.3 | mt |
| 14/06/2017 | Grass | Spread Organic Manure | Organic FYM | 14.7 | mt |
| 26/04/2018 | Grass | Spread Fertiliser | Yara Extran | 716 | kg |
| 01/05/2018 | Grass | Spread Fertiliser | TSP | 252 | kg |
| 31/05/2018 | Grass | Spread Fertiliser | Yara Extran | 750 | kg |
| 03/07/2018 | Grass | Spread Fertiliser | Yara Extran | 752 | kg |
| 27/03/2019 | Grass | Spread Fertiliser | MultiCut Sulphur 23-4-13-7 | 2276 | kg |
| 29/04/2019 | Grass | Spread Fertiliser | MultiCut Sulphur 23-4-13-7 | 1098 | kg |
| 29/05/2019 | Grass | Spread Fertiliser | MultiCut Sulphur 23-4-13-7 | 1116 | kg |
| 21/06/2019 | Grass | Apply Pesticide | Spray Herbicides | | l |
| 26/08/2019 | Grass | Apply Pesticide | Glyphosate |  | l |
| 04/09/2019 | Grass | Spread Organic Manure | Organic FYM | 109 | mt |
| 09/09/2019 | Arable | Plough |  |  |  |
| 10/09/2019 | Arable | Plough |  |  |  |
| 11/09/2019 | Arable | Plough |  |  |  |
| 13/09/2019 | Arable | Plough |  |  |  |
| 15/09/2019 | Arable | Cambridge/Ring roll |  |  |  |
| 25/09/2019 | Arable | Spread Fertiliser | TSP | 1020 | kg |
| 02/10/2019 | Arable | Spread Fertiliser | Lime | 46 | kg |
| 02/10/2019 | Arable | Drill Seed | Wheat (Crusoe) | 1217 | kg |
| 04/10/2019 | Arable | Cambridge/Ring roll |  |  |  |

Table A3: Land use and management history for field-scale catchment 3 on the NWFP.

| **Event Date** | **Land cover** | **Field Operation** | **Application** | **Total application** | **Units** |
| --- | --- | --- | --- | --- | --- |
| 08/04/2016 | Grass | Spread Fertiliser | 20-8-12-7 | 1002 | kg |
| 18/04/2016 | Grass | Flat roll |  |  |  |
| 23/05/2016 | Grass | Spreading grass |  |  |  |
| 23/05/2016 | Grass | Mow |  |  |  |
| 24/05/2016 | Grass | Rowing up |  |  |  |
| 24/05/2016 | Grass | Silage pick up |  |  |  |
| 25/05/2016 | Grass | Spread Organic Manure | Organic FYM | 39.2 | mt |
| 26/05/2016 | Grass | Spread Organic Manure | Organic FYM | 14.7 | mt |
| 21/06/2016 | Grass | Spread Fertiliser | Nitram | 300 | kg |
| 19/07/2016 | Grass | Spread Fertiliser | Nitram | 304 | kg |
| 05/04/2017 | Grass | Spread Fertiliser | Nitram | 310 | kg |
| 04/05/2017 | Grass | Spread Fertiliser | Nitram | 165 | kg |
| 05/05/2017 | Grass | Flat roll |  |  |  |
| 22/05/2017 | Grass | Mow |  |  |  |
| 22/05/2017 | Grass | Spreading grass |  |  |  |
| 23/05/2017 | Grass | Rowing up |  |  |  |
| 24/05/2017 | Grass | Round baling |  |  |  |
| 24/05/2017 | Grass | Drain spray | Roundup Biactive | | l |
| 09/06/2017 | Grass | Spread Fertiliser | Nitram | 316 | kg |
| 27/06/2017 | Grass | Drain trim |  |  |  |
| 04/07/2017 | Grass | Spread Fertiliser | Nitram | 306 | kg |
| 22/08/2017 | Grass | Mow |  |  |  |
| 22/08/2017 | Grass | Spreading grass |  |  |  |
| 23/08/2017 | Grass | Rowing up |  |  |  |
| 24/08/2017 | Grass | Round baling |  |  |  |
| 25/04/2018 | Grass | Spread Fertiliser | SilageBooster(20-4.5-14.5+7.5%) | 938 | kg |
| 30/04/2018 | Grass | Spread Fertiliser | TSP | 245 | kg |
| 01/05/2018 | Grass | Spread Fertiliser | MOP | 95 | kg |
| 02/05/2018 | Grass | Flat roll |  |  |  |
| 23/05/2018 | Grass | Mow |  |  |  |
| 23/05/2018 | Grass | Rowing up |  |  |  |
| 24/05/2018 | Grass | Silage pick up |  |  |  |
| 31/05/2018 | Grass | Spread Fertiliser | Yara Extran | 230 | kg |
| 05/06/2018 | Grass | Drain spray | Roundup Biactive | | l |
| 19/06/2018 | Grass | Drain trim |  |  |  |
| 03/07/2018 | Grass | Spread Fertiliser | Yara Extran | 299 | kg |
| 13/08/2018 | Grass | Spread Fertiliser | Yara Extran | 264 | kg |
| 17/08/2018 | Grass | Lime | Lime | 978 | kg |
| 27/03/2019 | Grass | Spread Fertiliser | MultiCut Sulphur 23-4-13-7 | 915 | kg |
| 28/03/2019 | Grass | Flat roll |  |  |  |
| 29/04/2019 | Grass | Spread Fertiliser | MultiCut Sulphur 23-4-13-7 | 421 | kg |
| 20/05/2019 | Grass | Mow |  |  |  |
| 21/05/2019 | Grass | Spreading grass |  |  |  |
| 21/05/2019 | Grass | Rowing up |  |  |  |
| 21/05/2019 | Grass | Silage pick up |  |  |  |
| 29/05/2019 | Grass | Spread Fertiliser | MultiCut Sulphur 23-4-13-7 | 423 | kg |
| 10/07/2019 | Grass | Drain trim |  |  |  |
| 20/08/2019 | Grass | Mow |  |  |  |
| 21/08/2019 | Grass | Rowing up |  |  |  |
| 21/08/2019 | Grass | Silage pick up |  |  |  |
| 26/08/2019 | Grass | Apply Pesticide | Glyphosate |  | l |
| 03/09/2019 | Grass | Spread Organic Manure | Organic FYM | 40 | mt |
| 05/09/2019 | Arable | Plough |  |  |  |
| 14/09/2019 | Arable | Plough |  |  |  |
| 23/09/2019 | Arable | Spread Fertiliser | Lime |  | kg |
| 24/09/2019 | Arable | Spread Fertiliser | TSP | 414 | kg |
| 02/10/2019 | Arable | Drill Seed | Wheat (Crusoe) | 489.3 | kg |
| 04/10/2019 | Arable | Cambridge/Ring roll |  |  |  |

Table A4: Land use and management history for field-scale catchment 5 on the NWFP.

| **Event Date** | **Land cover** | **Field Operation** | **Application** | **Total application** | **Units** |
| --- | --- | --- | --- | --- | --- |
| 13/04/2016 | Grass | Spread Fertiliser | Nitram | 410 | kg |
| 12/05/2016 | Grass | Spread Fertiliser | Nitram | 452 | kg |
| 15/06/2016 | Grass | Spread Fertiliser | Nitram | 438 | kg |
| 19/07/2016 | Grass | Spread Fertiliser | Nitram | 452 | kg |
| 28/07/2016 | Grass | Drain trim |  |  |  |
| 04/08/2016 | Grass | Spreading grass |  |  |  |
| 04/08/2016 | Grass | Mow |  |  |  |
| 06/08/2016 | Grass | Rowing up |  |  |  |
| 06/08/2016 | Grass | Round baling |  |  |  |
| 22/08/2016 | Grass | Spread Organic Manure | Organic FYM | 24.5 | mt |
| 22/08/2016 | Grass | Spread Organic Manure | Organic FYM | 4.9 | mt |
| 23/08/2016 | Grass | Spread Organic Manure | Organic FYM | 44.1 | mt |
| 24/08/2016 | Grass | Spread Organic Manure | Organic FYM | 9.8 | mt |
| 14/10/2016 | Grass | Drain burn |  |  |  |
| 12/04/2017 | Grass | Spread Fertiliser | Nitram | 440 | kg |
| 09/05/2017 | Grass | Spread Fertiliser | Nitram | 457 | kg |
| 24/05/2017 | Grass | Drain spray | Roundup Biactive | | l |
| 09/06/2017 | Grass | Spread Fertiliser | Nitram | 457 | kg |
| 16/06/2017 | Grass | Apply Pesticide | Spray Herbicides | 4 | l |
| 28/06/2017 | Grass | Drain trim |  |  |  |
| 25/04/2018 | Grass | Spread Fertiliser | SilageBooster(20-4.5-14.5+7.5%) | 1550 | kg |
| 30/04/2018 | Grass | Spread Fertiliser | TSP | 348 | kg |
| 08/05/2018 | Grass | Flat roll |  |  |  |
| 23/05/2018 | Grass | Mow |  |  |  |
| 23/05/2018 | Grass | Rowing up |  |  |  |
| 24/05/2018 | Grass | Silage pick up |  |  |  |
| 31/05/2018 | Grass | Spread Fertiliser | Yara Extran | 438 | kg |
| 05/06/2018 | Grass | Drain spray | Roundup Biactive | | l |
| 20/06/2018 | Grass | Drain trim |  |  |  |
| 20/06/2018 | Grass | Drain trim |  |  |  |
| 13/08/2018 | Grass | Spread Fertiliser | Yara Extran | 404 | kg |
| 17/08/2018 | Grass | Lime | Lime | 2415 | kg |
| 28/03/2019 | Grass | Spread Fertiliser | Nitram | 395 | kg |
| 03/05/2019 | Grass | Spread Fertiliser | Nitram | 429 | kg |
| 30/05/2019 | Grass | Spread Fertiliser | Nitram | 387 | kg |
| 04/06/2019 | Grass | Spread Fertiliser | Kieserite | 240 | kg |
| 09/07/2019 | Grass | Drain trim |  |  |  |
| 20/08/2019 | Grass | Mow |  |  |  |
| 21/08/2019 | Grass | Rowing up |  |  |  |
| 22/08/2019 | Grass | Round baling |  |  |  |
| 13/09/2019 | Grass | Spread Organic Manure | Organic FYM | 30 | mt |
| 16/09/2019 | Grass | Spread Organic Manure | Organic FYM | 30 | mt |

Table A5: Land use and management history for field-scale catchment 8 on the NWFP.

| **Event Date** | **Land**  **cover** | **Field Operation** | **Application** | **Total application** | **Units** |
| --- | --- | --- | --- | --- | --- |
| 15/06/2016 | Grass | Top |  |  |  |
| 14/07/2016 | Grass | Apply Pesticide | Pinnacle and Spruce mix | 10.78 | l |
| 01/08/2016 | Grass | Drain trim |  |  |  |
| 02/08/2016 | Grass | Drain trim |  |  |  |
| 08/08/2016 | Grass | Rowing up |  |  |  |
| 08/08/2016 | Grass | Mow |  |  |  |
| 08/08/2016 | Grass | Round baling |  |  |  |
| 30/08/2016 | Grass | Spread Organic Manure | Organic FYM | 19.6 | mt |
| 31/08/2016 | Grass | Spread Organic Manure | Organic FYM | 53.9 | mt |
| 05/09/2016 | Grass | Spread Organic Manure | Organic FYM | 19.6 | mt |
| 17/10/2016 | Grass | Drain burn |  |  |  |
| 11/04/2017 | Grass | Apply Pesticide | Spray Herbicides | 69.9 | l |
| 23/05/2017 | Grass | Drain spray | Roundup Biactive | | l |
| 30/06/2017 | Grass | Drain trim |  |  |  |
| 21/08/2017 | Grass | Mow |  |  |  |
| 22/08/2017 | Grass | Spreading grass |  |  |  |
| 23/08/2017 | Grass | Rowing up |  |  |  |
| 23/08/2017 | Grass | Round baling |  |  |  |
| 26/04/2018 | Grass | Spread Fertiliser | Yara Extran | 460 | kg |
| 30/04/2018 | Grass | Spread Fertiliser | TSP | 628 | kg |
| 01/05/2018 | Grass | Spread Fertiliser | MOP | 504 | kg |
| 02/05/2018 | Grass | Flat roll |  |  |  |
| 23/05/2018 | Grass | Mow |  |  |  |
| 23/05/2018 | Grass | Rowing up |  |  |  |
| 24/05/2018 | Grass | Silage pick up |  |  |  |
| 06/06/2018 | Grass | Drain spray | Roundup Biactive | | l |
| 21/06/2018 | Grass | Drain trim |  |  |  |
| 30/07/2018 | Grass | Drain burn |  |  |  |
| 26/04/2019 | Grass | Flat roll |  |  |  |
| 04/06/2019 | Grass | Spread Fertiliser | Kieserite | 229 | kg |
| 05/07/2019 | Grass | Drain trim |  |  |  |
| 23/07/2019 | Grass | Spread Fertiliser | Nitram | 123 | kg |
| 20/08/2019 | Grass | Mow |  |  |  |
| 22/08/2019 | Grass | Rowing up |  |  |  |
| 22/08/2019 | Grass | Round baling |  |  |  |
| 20/09/2019 | Grass | Spread Organic Manure | Organic FYM | 25 | mt |
| 23/09/2019 | Grass | Spread Organic Manure | Organic FYM | 25 | mt |
| 04/09/2014 | Grass | Drain spray | Roundup Biactive | | l |
| 08/04/2015 | Grass | Flat roll |  |  |  |
| 15/06/2015 | Grass | Spread Fertiliser | TSP | 298 | kg |
| 14/07/2015 | Grass | Drain burn |  |  |  |
| 21/07/2015 | Grass | Drain trim |  |  |  |
| 19/04/2016 | Grass | Flat roll |  |  |  |
| 19/04/2016 | Grass | Spread Fertiliser | 0-20-30 | 437 | kg |
| 09/05/2016 | Grass | Apply Pesticide | Pinnacle and Spruce mix | 6.78 | l |
| 03/06/2016 | Grass | Mow |  |  |  |
| 06/06/2016 | Grass | Spread Organic Manure | Organic FYM | 44.1 | mt |
| 07/06/2016 | Grass | Spread Organic Manure | Organic FYM | 9.8 | mt |
| 21/06/2016 | Grass | Spread Fertiliser | MOP | 345 | kg |
| 21/06/2016 | Grass | Lime | Calcifert lime | 1200 | kg |
| 02/08/2016 | Grass | Drain trim |  |  |  |
| 17/10/2016 | Grass | Drain burn |  |  |  |
| 04/04/2017 | Grass | Apply Pesticide | Spray Herbicides | 60.9 | l |
| 23/05/2017 | Grass | Drain spray | Roundup Biactive | | l |
| 03/07/2017 | Grass | Drain trim |  |  |  |
| 26/07/2017 | Grass | Apply Pesticide | Spray Herbicides | | l |
| 21/08/2017 | Grass | Mow |  |  |  |
| 22/08/2017 | Grass | Spreading grass |  |  |  |
| 22/08/2017 | Grass | Rowing up |  |  |  |
| 23/08/2017 | Grass | Round baling |  |  |  |
| 26/04/2018 | Grass | Spread Fertiliser | Yara Extran | 300 | kg |
| 30/04/2018 | Grass | Spread Fertiliser | TSP | 384 | kg |
| 01/05/2018 | Grass | Spread Fertiliser | MOP | 123 | kg |
| 02/05/2018 | Grass | Flat roll |  |  |  |
| 23/05/2018 | Grass | Mow |  |  |  |
| 23/05/2018 | Grass | Rowing up |  |  |  |
| 24/05/2018 | Grass | Silage pick up |  |  |  |
| 06/06/2018 | Grass | Drain spray | Roundup Biactive | | l |
| 21/06/2018 | Grass | Drain trim |  |  |  |
| 30/07/2018 | Grass | Drain burn |  |  |  |
| 30/07/2018 | Grass | Drain burn |  |  |  |
| 04/06/2019 | Grass | Spread Fertiliser | Kieserite | 150 | kg |
| 11/07/2019 | Grass | Drain trim |  |  |  |
| 23/07/2019 | Grass | Spread Fertiliser | Nitram | 238 | kg |
| 20/08/2019 | Grass | Mow |  |  |  |
| 22/08/2019 | Grass | Rowing up |  |  |  |
| 22/08/2019 | Grass | Round baling |  |  |  |
| 19/09/2019 | Grass | Spread Organic Manure | Organic FYM | 15 | mt |
| 20/09/2019 | Grass | Spread Organic Manure | Organic FYM | 15 | mt |

**Table A6.** The 19 global climate models (GCMs) from the CMIP5 multi-model ensemble used in the present study.

| **No.** | **GCM** | **Research centre** | **Country** | **Grid resolution** | **Reference^†^** |
| --- | --- | --- | --- | --- | --- |
| 1 | ACCESS1-3 | The Centre for Australian Weather and Climate Research | Australia | 1.25° x 1.88° | ^1^ |
| 2 | BCC-CSM1-1 | Beijing Climate Center | China | 2.77° x 2.81° | ^2^ |
| 3 | CanESM2 | Canadian Centre for Climate Modelling and Analysis | Canada | 2.77° x 2.81° | ^3^ |
| 4 | CMCC-CM | Centro Euro-Mediterraneo sui Cambiamenti Climatici | Italy | 0.74° x 0.75° | ^4^ |
| 5 | CNRM-CM5 | CNRM-GAME and Cerfacs | France | 1.40° x 1.40° | ^5^ ^6^ |
| 6 | CSIRO-Mk3.6 | Australia's Commonwealth Scientific and Industrial Research Organisation | Australia | 1.85° x 1.88° | ^7^ |
| 7 | EC-EARTH | EC-Earth consortium | Europe | 1.125° x 1.125° | ^8^ |
| 8 | GFDL-CM3 | Geophysical Fluid Dynamics Laboratory | USA | 2.00° x 2.50° | ^9^ |
| 9 | GISS-E2-R-CC | Goddard Institute for Space Studies | USA | 2.00° x 2.50° | ^10^ |
| 10 | HadGEM2-ES | UK Meteorological Office | UK | 1.25° x 1.88° | ^11,12^ |
| 11 | INMCM4 | Institute for Numerical Mathematics | Russia | 1.50° x 20° | ^13,14^ |
| 12 | IPSL-CM5A-MR | Institute Pierre Simon Laplace | France | 1.27° x 2.50° | ^15^ |
| 13 | MIROC5 | University of Tokyo, National Institute for Environnemental Studies, Japan Agency for Marine-Earth Science and Technology | Japan | 1.39° x 1.41° | ^16,17^ |
| 14 | MIROC-ESM | University of Tokyo, National Institute for Environnemental Studies, Japan Agency for Marine-Earth Science and Technology | Japan | 2.77° x 2.81° | ^17^ |
| 15 | MPI-ESM-MR | Max-Planck Institute for Meteorology | Germany | 1.85° x 1.88° | ^18,19^ |
| 16 | MRI-CGCM3 | Meteorological Research Institute | Japan | 1.11° x 1.13° | ^20^ |
| 17 | NCAR-CCSM4 | National Centre for Atmospheric Research | USA | 0.94° x 1.25° | ^21,22^ |
| 18 | NCAR-CESM1-CAM5 | National Centre for Atmospheric Research | USA | 0.94° x 1.25° | ^22^ |
| 19 | NorESM1-M | Norwegian Climate Centre | Norway | 1.90° x 2.50° | ^23,24^ |

**^†^References**

1 Collier, M. & Uhe, P. CMIP5 datasets from the ACCESS1.0 and ACCESS1.3 coupled climate models. CAWCR Technical Report No. 059. The Centre for Australian Weather and Climate Researc, Australia. (2012).

2 Zhang, L., Wu, T. W., Xin, X. G., Dong, M. & Wang, Z. Z. Projections of annual mean air temperature and precipitation over the globe and in China during the 21st century by the BCC Climate System Model BCC_CSM1.0. *Acta Meteorol. Sin.* **26**, 362-375, doi:10.1007/s13351-012-0308-8 (2012).

3 Chylek, P., Li, J., Dubey, M., Wang, M. & Lesins, G. Observed and model simulated 20th century Arctic temperature variability: Canadian Earth System Model CanESM2. *Atmospheric Chemistry and Physics Discussions* **11**, 22893-22907, doi:10.5194/acpd-11-22893-2011 (2011).

4 Bellucci, A. *et al.* Decadal climate predictions with a coupled OAGCM initialized with oceanic reanalyses. *Clim. Dyn.* **40**, 1483-1497, doi:10.1007/s00382-012-1468-z (2013).

5 Voldoire, A. *et al.* The CNRM-CM5.1 global climate model: description and basic evaluation. *Clim. Dyn.* **40**, 2091-2121, doi:10.1007/s00382-011-1259-y (2013).

6 Voldoire, A. *et al.* The CNRM-CM5.1 global climate model: description and basic evaluation. *Climate Dynamics* **40**, 2091-2121, doi:10.1007/s00382-011-1259-y (2013).

7 Jeffrey, S. *et al.* Australia's CMIP5 submission using the CSIRO-Mk3.6 model. *Aust. Meteorol. Oceanogr. J.* **63**, 1-13 (2013).

8 Hazeleger, W. *et al.* EC-Earth V2.2: description and validation of a new seamless earth system prediction model. *Clim. Dyn.* **39**, 2611-2629, doi:10.1007/s00382-011-1228-5 (2012).

9 Griffies, S. M. *et al.* The GFDL CM3 Coupled Climate Model: Characteristics of the Ocean and Sea Ice Simulations. *J. Clim.* **24**, 3520-3544, doi:10.1175/2011jcli3964.1 (2011).

10 Chandler, M. A., Sohl, L. E., Jonas, J. A., Dowsett, H. J. & Kelley, M. Simulations of the mid-Pliocene Warm Period using two versions of the NASA/GISS ModelE2-R Coupled Model. *Geosci. Model Dev.* **6**, 517-531, doi:10.5194/gmd-6-517-2013 (2013).

11 Collins, W. J. *et al.* Development and evaluation of an Earth-System model-HadGEM2. *Geosci. Model Dev.* **4**, 1051-1075, doi:10.5194/gmd-4-1051-2011 (2011).

12 Jones, C. D. *et al.* The HadGEM2-ES implementation of CMIP5 centennial simulations. *Geosci. Model Dev.* **4**, 543-570, doi:10.5194/gmd-4-543-2011 (2011).

13 Volodin, E. M., Diansky, N. A. & Gusev, A. V. Simulation and prediction of climate changes in the 19th to 21st centuries with the Institute of Numerical Mathematics, Russian Academy of Sciences, model of the Earth's climate system. *Izv. Atmos. Ocean. Phys.* **49**, 347-366, doi:10.1134/s0001433813040105 (2013).

14 Yurova, A. Y. & Volodin, E. M. Coupled simulation of climate and vegetation dynamics. *Izv. Atmos. Ocean. Phys.* **47**, 531-539, doi:10.1134/s0001433811050124 (2011).

15 Dufresne, J. L. *et al.* Climate change projections using the IPSL-CM5 Earth System Model: from CMIP3 to CMIP5. *Clim. Dyn.* **40**, 2123-2165, doi:10.1007/s00382-012-1636-1 (2013).

16 Mochizuki, T. *et al.* Decadal Prediction Using a Recent Series of MIROC Global Climate Models. *J. Meteorol. Soc. Jpn.* **90A**, 373-383, doi:10.2151/jmsj.2012-A22 (2012).

17 Watanabe, S. *et al.* MIROC-ESM 2010: model description and basic results of CMIP5-20c3m experiments. *Geosci. Model Dev.* **4**, 845-872, doi:10.5194/gmd-4-845-2011 (2011).

18 Brovkin, V. *et al.* Evaluation of vegetation cover and land-surface albedo in MPI-ESM CMIP5 simulations. *J. Adv. Model. Earth Syst.* **5**, 48-57, doi:10.1029/2012ms000169 (2013).

19 Schmidt, H. *et al.* Response of the middle atmosphere to anthropogenic and natural forcings in the CMIP5 simulations with the Max Planck Institute Earth system model. *J. Adv. Model. Earth Syst.* **5**, 98-116, doi:10.1002/jame.20014 (2013).

20 Tsujino, H. *et al.* Simulating present climate of the global ocean-ice system using the Meteorological Research Institute Community Ocean Model (MRI.COM): simulation characteristics and variability in the Pacific sector. *J. Oceanogr.* **67**, 449-479, doi:10.1007/s10872-011-0050-3 (2011).

21 Jahn, A. & Holland, M. M. Implications of Arctic sea ice changes for North Atlantic deep convection and the meridional overturning circulation in CCSM4-CMIP5 simulations. *Geophys. Res. Lett.* **40**, 1206-1211, doi:10.1002/grl.50183 (2013).

22 Meehl, G. A. *et al.* Climate Change Projections in CESM1(CAM5) Compared to CCSM4. *J. Clim.* **26**, 6287-6308, doi:10.1175/jcli-d-12-00572.1 (2013).

23 Bentsen, M. *et al.* The Norwegian Earth System Model, NorESM1-M - Part 1: Description and basic evaluation of the physical climate. *Geosci. Model Dev.* **6**, 687-720, doi:10.5194/gmd-6-687-2013 (2013).

24 Iversen, T. *et al.* The Norwegian Earth System Model, NorESM1-M - Part 2: Climate response and scenario projections. *Geosci. Model Dev.* **6**, 389-415, doi:10.5194/gmd-6-389-2013 (2013).

**Table A7A.** Summary statistics (October – March, inclusive) for field-scale flow rates (l s^-1^) on the NWFP.

|  | **Field 2** | | |  | **Field 3** | | |  | **Field 5** | | |  | **Field 8** | | |  |
| --- | --- | --- | --- | --- | --- | --- | --- | --- | --- | --- | --- | --- | --- | --- | --- | --- |
| **Month Year** | **Mean** | **Median** | **P95*** | **IQR^+^** | **Mean** | **Median** | **P95*** | **IQR^+^** | **Mean** | **Median** | **P95*** | **IQR^+^** | **Mean** | **Median** | **P95*** | **IQR^+^** |
| Nov 16 | 0.31 | 0.02 | 0.99 | 0.05 | 0.45 | 0.03 | 1.74 | 0.16 | 0.33 | 0.03 | 0.94 | 0.10 | 0.38 | 0.00 | 0.99 | 0.03 |
| Dec 16 | 0.05 | 0.03 | 0.28 | 0.04 | 0.12 | 0.09 | 0.64 | 0.08 | 0.03 | 0.02 | 0.07 | 0.03 | 0.03 | 0.00 | 0.09 | 0.05 |
| Jan 17 | 0.32 | 0.14 | 1.04 | 0.18 | 0.60 | 0.28 | 2.41 | 0.30 | 0.30 | 0.16 | 0.68 | 0.19 | 0.32 | 0.10 | 0.81 | 0.17 |
| Feb 17 | 0.56 | 0.10 | 1.60 | 0.28 | 0.78 | 0.25 | 1.99 | 0.43 | 0.71 | 0.25 | 1.81 | 0.41 | 0.88 | 0.12 | 2.76 | 0.24 |
| Mar 17 | 0.64 | 0.18 | 2.43 | 0.40 | 0.89 | 0.43 | 2.58 | 0.54 | 0.84 | 0.43 | 2.34 | 0.49 | 0.86 | 0.23 | 3.20 | 0.43 |
| Oct 17 | 0.28 | 0.05 | 0.77 | 0.04 | 0.33 | 0.12 | 0.64 | 0.07 | 0.16 | 0.03 | 0.36 | 0.01 | 0.30 | 0.05 | 0.77 | 0.11 |
| Nov 17 | 0.74 | 0.10 | 4.02 | 0.21 | 0.90 | 0.23 | 3.83 | 0.47 | 0.69 | 0.12 | 3.46 | 0.37 | 0.80 | 0.09 | 4.21 | 0.28 |
| Dec 17 | 1.83 | 0.23 | 9.01 | 0.58 | 1.96 | 0.33 | 8.71 | 0.63 | 2.08 | 0.36 | 9.30 | 0.60 | 2.00 | 0.23 | 9.60 | 0.52 |
| Jan 18 | 2.21 | 0.73 | 6.93 | 1.61 | 2.21 | 0.85 | 7.35 | 1.50 | 2.47 | 0.90 | 7.85 | 1.46 | 2.14 | 0.43 | 7.85 | 1.48 |
| Feb 18 | 0.65 | 0.23 | 1.87 | 0.22 | 0.79 | 0.36 | 2.11 | 0.33 | 0.76 | 0.33 | 1.74 | 0.33 | 0.57 | 0.12 | 1.74 | 0.13 |
| Mar 18 | 2.42 | 0.64 | 10.95 | 1.05 | 2.67 | 0.77 | 12.51 | 1.07 | 2.71 | 0.77 | 12.11 | 1.09 | 2.71 | 0.53 | 13.94 | 1.27 |
| Oct 18 | 0.08 | 0.00 | 0.64 | 0.00 | 0.11 | 0.05 | 0.36 | 0.12 | 0.08 | 0.03 | 0.28 | 0.07 | 0.02 | 0.00 | 0.03 | 0.00 |
| Nov 18 | 1.47 | 0.23 | 5.25 | 1.01 | 1.27 | 0.25 | 4.92 | 0.81 | 1.53 | 0.28 | 5.25 | 0.96 | 1.60 | 0.06 | 6.67 | 0.50 |
| Dec 18 | 2.31 | 1.34 | 7.06 | 2.07 | 2.38 | 1.34 | 6.93 | 1.87 | 2.60 | 1.18 | 8.75 | 1.95 | 2.95 | 1.04 | 11.59 | 2.25 |
| Jan 19 | 0.62 | 0.20 | 1.82 | 0.45 | 0.84 | 0.28 | 2.27 | 0.71 | 0.64 | 0.20 | 1.81 | 0.58 | 0.54 | 0.15 | 1.69 | 0.26 |
| Feb 19 | 1.53 | 0.64 | 5.03 | 1.16 | 1.34 | 0.36 | 4.71 | 1.25 | 1.47 | 0.53 | 4.50 | 0.98 | 1.27 | 0.25 | 4.99 | 0.58 |
| Oct 19 | 0.68 | 0.21 | 3.35 | 0.42 | 0.83 | 0.23 | 3.37 | 0.71 | 0.71 | 0.23 | 3.11 | 0.43 | 1.31 | 0.36 | 6.80 | 0.53 |
| Nov 19 | 2.22 | 1.13 | 8.13 | 1.42 | 3.25 | 1.68 | 11.76 | 2.14 | 2.56 | 0.99 | 9.60 | 1.25 | 2.65 | 0.74 | 11.60 | 1.60 |
| Dec 19 | 2.95 | 0.94 | 13.00 | 1.88 | 3.25 | 1.08 | 14.18 | 1.66 | 3.35 | 1.18 | 13.80 | 2.23 | 3.26 | 0.68 | 14.96 | 2.31 |
| Jan 20 | 1.82 | 0.73 | 6.32 | 0.95 | 1.98 | 0.77 | 6.16 | 0.70 | 1.79 | 0.81 | 4.82 | 0.72 | 1.78 | 0.40 | 5.81 | 0.62 |
| Feb 20 | 4.08 | 1.13 | 19.17 | 2.50 | 4.66 | 1.45 | 20.87 | 2.63 | 4.93 | 1.93 | 19.40 | 2.50 | 4.96 | 1.23 | 23.85 | 3.33 |
| Mar 20 | 2.41 | 0.81 | 8.27 | 1.27 | 2.77 | 1.13 | 9.45 | 1.28 | 2.78 | 1.45 | 7.72 | 1.45 | 2.69 | 0.77 | 9.92 | 1.38 |

* P95: 95^th^ percentile; ^+^ IQR: Inter-Quartile Range

**Table A7B.** Summary statistics (October – March, inclusive) for field-scale runoff nitrate concentrations (mg l^-1^) on the NWFP.

|  | **Field 2** |  |  |  | **Field 3** |  |  |  | **Field 5** |  |  |  | **Field 8** |  |  |  |
| --- | --- | --- | --- | --- | --- | --- | --- | --- | --- | --- | --- | --- | --- | --- | --- | --- |
| **Month Year** | **Mean** | **Median** | **P95** | **IQR^+^** | **Mean** | **Median** | **P95** | **IQR^+^** | **Mean** | **Median** | **P95** | **IQR^+^** | **Mean** | **Median** | **P95** | **IQR^+^** |
| Nov 16 | 2.2 | 2.2 | 2.8 | 0.5 | 3.1 | 2.8 | 5.0 | 0.6 | 5.4 | 5.7 | 7.3 | 1.8 | 3.0 | 3.2 | 4.1 | 1.7 |
| Dec 16 | 1.6 | 1.6 | 2.2 | 0.3 | 1.6 | 1.5 | 2.1 | 0.2 | 4.7 | 5.0 | 5.3 | 1.0 | 2.0 | 2.1 | 2.4 | 0.5 |
| Jan 17 | 1.7 | 1.7 | 2.5 | 0.6 | 2.9 | 2.8 | 4.5 | 1.0 | 4.9 | 5.3 | 5.9 | 1.6 | 5.1 | 4.6 | 8.4 | 2.7 |
| Feb 17 | 1.7 | 1.7 | 2.0 | 0.4 | 2.5 | 2.6 | 3.0 | 0.5 | 4.4 | 4.8 | 5.2 | 1.2 | 3.1 | 3.5 | 4.0 | 1.1 |
| Mar 17 | 1.3 | 1.2 | 1.9 | 0.4 | 2.0 | 2.1 | 2.5 | 0.5 | 3.6 | 3.9 | 4.7 | 1.4 | 1.7 | 1.7 | 2.2 | 0.7 |
| Oct 17 | 1.6 | 1.4 | 2.2 | 0.5 | 1.6 | 1.3 | 2.3 | 1.0 | 2.7 | 2.7 | 3.5 | 0.7 | 1.3 | 1.3 | 1.7 | 0.3 |
| Nov 17 | 1.7 | 1.6 | 2.3 | 0.5 | 1.5 | 1.4 | 2.1 | 0.6 |  |  |  |  | 1.2 | 1.2 | 1.8 | 1.0 |
| Dec 17 | 1.6 | 1.8 | 2.2 | 0.8 | 1.9 | 2.1 | 2.5 | 0.6 | 4.0 | 4.4 | 6.2 | 2.7 | 1.4 | 1.5 | 1.9 | 0.9 |
| Jan 18 | 1.4 | 1.3 | 2.4 | 1.0 | 1.5 | 1.4 | 2.5 | 1.3 | 3.2 | 3.1 | 5.4 | 2.8 | 0.8 | 0.8 | 1.5 | 0.6 |
| Feb 18 | 1.7 | 1.8 | 2.2 | 0.6 | 1.7 | 1.9 | 2.3 | 0.6 | 3.9 | 4.2 | 5.0 | 1.7 | 1.2 | 1.3 | 1.6 | 0.6 |
| Mar 18 | 1.2 | 1.2 | 2.0 | 0.6 | 1.0 | 0.9 | 1.8 | 0.6 | 2.6 | 2.6 | 5.0 | 1.7 | 0.8 | 0.8 | 1.4 | 0.5 |
| Oct 18 | 8.6 | 8.4 | 11.6 | 2.5 | 8.2 | 8.3 | 9.9 | 0.8 | 5.2 | 4.9 | 7.7 | 1.8 | 3.0 | 2.8 | 5.0 | 0.4 |
| Nov 18 | 4.3 | 3.9 | 7.3 | 0.4 | 6.5 | 6.9 | 10.4 | 4.3 | 6.3 | 6.8 | 8.0 | 2.3 | 2.0 | 2.2 | 3.1 | 0.8 |
| Dec 18 | 1.8 | 1.8 | 2.3 | 0.6 | 2.2 | 2.1 | 2.7 | 0.4 | 3.3 | 3.3 | 5.1 | 1.7 | 1.0 | 1.0 | 1.3 | 0.3 |
| Jan 19 | 2.4 | 2.4 | 2.7 | 0.4 | 3.6 | 3.5 | 4.9 | 1.1 | 4.4 | 4.6 | 5.7 | 1.0 | 1.2 | 1.3 | 1.5 | 0.3 |
| Feb 19 | 2.0 | 2.0 | 2.8 | 1.1 | 2.9 | 3.0 | 3.5 | 0.7 | 5.0 | 5.6 | 6.9 | 2.7 | 0.9 | 1.0 | 1.2 | 0.3 |
| Oct 19 | 13.6 | 13.3 | 19.4 | 5.5 | 12.0 | 12.6 | 14.3 | 3.0 | 2.9 | 2.9 | 4.4 | 1.5 | 1.4 | 1.3 | 2.0 | 0.3 |
| Nov 19 | 4.0 | 3.6 | 7.5 | 2.1 | 5.2 | 5.0 | 7.6 | 3.5 | 2.7 | 2.7 | 4.2 | 0.9 | 1.0 | 1.0 | 1.1 | 0.1 |
| Dec 19 | 2.4 | 2.4 | 4.8 | 1.5 | 3.4 | 3.2 | 5.9 | 1.9 | 2.1 | 1.9 | 4.1 | 1.4 | 0.8 | 0.8 | 1.1 | 0.3 |
| Jan 20 | 2.3 | 2.3 | 3.2 | 0.8 | 2.5 | 2.6 | 3.7 | 1.1 | 2.1 | 2.1 | 2.8 | 0.7 | 0.9 | 0.9 | 1.0 | 0.1 |
| Feb 20 | 1.8 | 1.7 | 3.2 | 0.9 | 1.7 | 1.5 | 3.2 | 0.8 |  |  |  |  | 0.7 | 0.7 | 1.0 | 0.4 |
| Mar 20 | 1.9 | 2.0 | 2.7 | 0.9 | 1.5 | 1.5 | 2.0 | 0.6 |  |  |  |  |  |  |  |  |

* P95: 95^th^ percentile; ^+^ IQR: Inter-Quartile Range and empty cells indicate that no valid samples are available

**Table A7C.** Summary statistics (October – March, inclusive) for field-scale runoff suspended sediment concentrations (mg l^-1^) on the NWFP.

|  | **Field 2** |  |  |  | **Field 3** |  |  |  | **Field 5** |  |  |  | **Field 8** |  |  |  |
| --- | --- | --- | --- | --- | --- | --- | --- | --- | --- | --- | --- | --- | --- | --- | --- | --- |
| **Month Year** | **Mean** | **Median** | **P95** | **IQR^+^** | **Mean** | **Median** | **P95** | **IQR^+^** | **Mean** | **Median** | **P95** | **IQR^+^** | **Mean** | **Median** | **P95** | **IQR^+^** |
| Nov 16 | 20.1 | 16.1 | 55.8 | 19.0 | 16.3 | 13.5 | 34.2 | 13.1 | 32.7 | 27.0 | 74.3 | 19.0 | 39.3 | 26.2 | 111.7 | 28.3 |
| Dec 16 | 10.8 | 7.7 | 25.3 | 31.0 | 7.2 | 6.0 | 13.6 | 1.9 | 43.8 | 30.5 | 115.7 | 31.0 | 17.2 | 9.3 | 41.7 | 15.8 |
| Jan 17 | 11.9 | 9.3 | 27.1 | 9.2 | 8.8 | 6.1 | 22.5 | 5.1 | 18.9 | 12.4 | 47.3 | 9.2 | 17.4 | 9.8 | 47.2 | 15.5 |
| Feb 17 | 11.8 | 9.0 | 24.3 | 6.7 | 11.2 | 5.9 | 40.1 | 6.9 | 17.7 | 10.7 | 45.5 | 6.7 | 17.0 | 8.6 | 49.9 | 17.2 |
| Mar 17 | 11.8 | 9.7 | 23.6 | 10.2 | 13.0 | 7.9 | 35.6 | 8.9 | 19.5 | 13.4 | 43.2 | 10.2 | 16.3 | 10.3 | 45.0 | 17.5 |
| Oct 17 | 25.2 | 20.4 | 51.2 | 28.7 | 12.2 | 6.4 | 39.8 | 7.2 | 39.9 | 30.0 | 96.7 | 28.7 | 36.1 | 28.2 | 78.9 | 40.4 |
| Nov 17 | 23.3 | 19.1 | 50.5 | 8.9 | 16.5 | 10.3 | 48.8 | 9.6 | 20.2 | 15.2 | 46.7 | 8.9 | 23.2 | 16.0 | 58.3 | 30.3 |
| Dec 17 | 15.7 | 9.1 | 45.2 | 8.0 | 20.1 | 9.0 | 56.9 | 11.2 | 18.0 | 11.1 | 41.8 | 8.0 | 23.1 | 8.3 | 80.0 | 19.9 |
| Jan 18 | 13.9 | 12.1 | 26.7 | 7.3 | 14.4 | 10.7 | 33.4 | 8.4 | 17.7 | 13.8 | 36.4 | 7.3 | 19.6 | 14.8 | 47.3 | 13.7 |
| Feb 18 | 9.7 | 7.3 | 19.4 | 3.1 | 10.3 | 7.2 | 23.2 | 1.9 | 13.5 | 10.8 | 22.3 | 3.1 | 13.1 | 6.9 | 39.2 | 7.5 |
| Mar 18 | 14.5 | 13.6 | 25.0 | 8.6 | 17.8 | 14.8 | 35.0 | 12.5 | 19.6 | 17.0 | 36.0 | 8.6 | 17.5 | 15.0 | 36.4 | 12.8 |
| Oct 18 | 27.8 | 22.7 | 56.0 | 11.8 | 12.0 | 8.9 | 27.0 | 8.2 | 26.5 | 24.4 | 43.7 | 11.8 | 26.7 | 25.9 | 44.1 | 11.6 |
| Nov 18 | 12.9 | 9.2 | 30.3 | 8.0 | 14.0 | 9.9 | 37.9 | 8.9 | 16.0 | 13.0 | 34.3 | 8.0 | 32.4 | 22.4 | 85.5 | 20.1 |
| Dec 18 | 11.1 | 9.2 | 21.6 | 4.0 | 10.1 | 7.8 | 21.8 | 5.6 | 14.4 | 11.5 | 23.8 | 4.0 | 13.6 | 10.3 | 31.3 | 9.3 |
| Jan 19 | 9.5 | 7.6 | 18.9 | 2.6 | 8.0 | 5.6 | 16.2 | 1.1 | 15.1 | 11.9 | 30.9 | 2.6 | 14.2 | 9.0 | 39.2 | 9.3 |
| Feb 19 | 10.3 | 7.8 | 22.1 | 2.2 | 8.5 | 5.3 | 23.4 | 3.0 | 12.0 | 10.0 | 19.8 | 2.2 | 12.1 | 7.5 | 31.7 | 8.4 |
| Oct 19 | 26.2 | 9.1 | 124.9 | 9.3 | 24.6 | 6.7 | 89.6 | 7.2 | 21.9 | 14.4 | 62.4 | 9.3 | 20.1 | 12.3 | 55.9 | 18.0 |
| Nov 19 | 59.8 | 19.8 | 263.1 | 8.1 | 76.3 | 10.6 | 390.3 | 20.9 | 19.9 | 13.6 | 44.3 | 8.1 | 14.6 | 10.1 | 35.7 | 10.2 |
| Dec 19 | 114.4 | 21.4 | 621.0 | 10.4 | 91.7 | 10.3 | 551.5 | 23.5 | 22.3 | 14.4 | 54.6 | 10.4 | 20.9 | 11.5 | 62.0 | 13.5 |
| Jan 20 | 83.9 | 14.9 | 387.3 | 5.0 | 53.9 | 10.5 | 220.8 | 9.6 | 18.7 | 13.0 | 42.5 | 5.0 | 17.5 | 9.5 | 43.4 | 8.5 |
| Feb 20 | 133.2 | 42.0 | 621.3 | 11.0 | 84.7 | 22.8 | 406.0 | 54.4 | 24.3 | 17.3 | 61.0 | 11.0 | 35.1 | 16.4 | 107.5 | 18.5 |
| Mar 20 | 68.5 | 25.5 | 228.3 | 6.8 | 44.1 | 14.4 | 152.4 | 21.3 | 22.6 | 14.3 | 47.9 | 6.8 | 17.6 | 11.9 | 39.0 | 8.5 |

* P95: 95th percentile; + IQR: Inter-Quartile Range

**
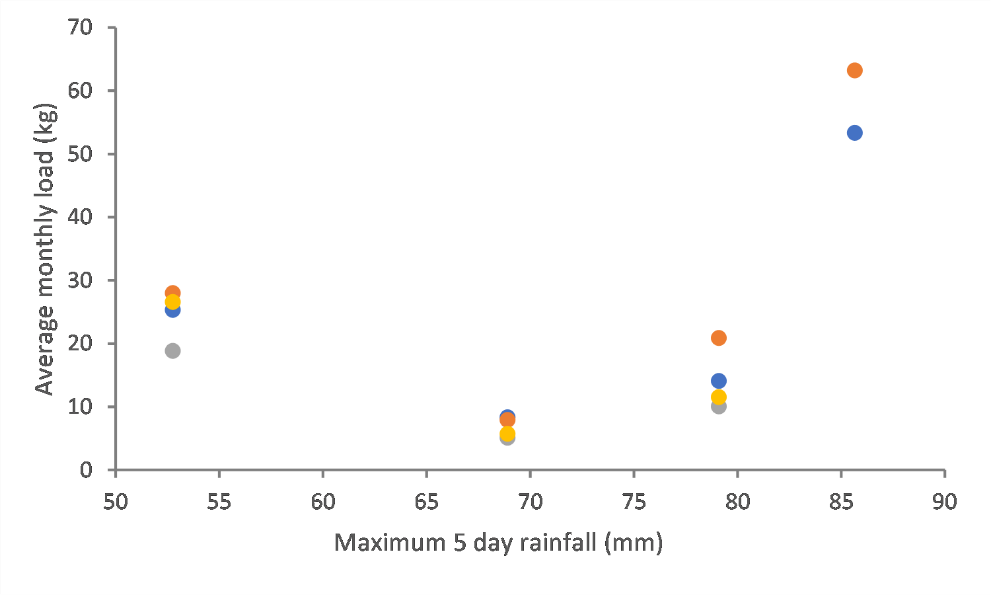
**

**
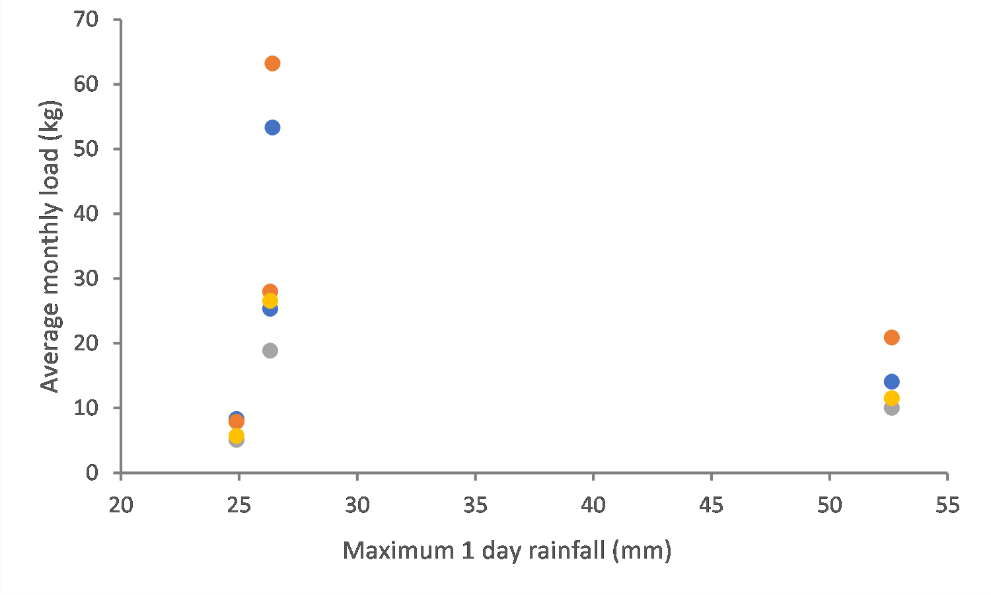
**

**
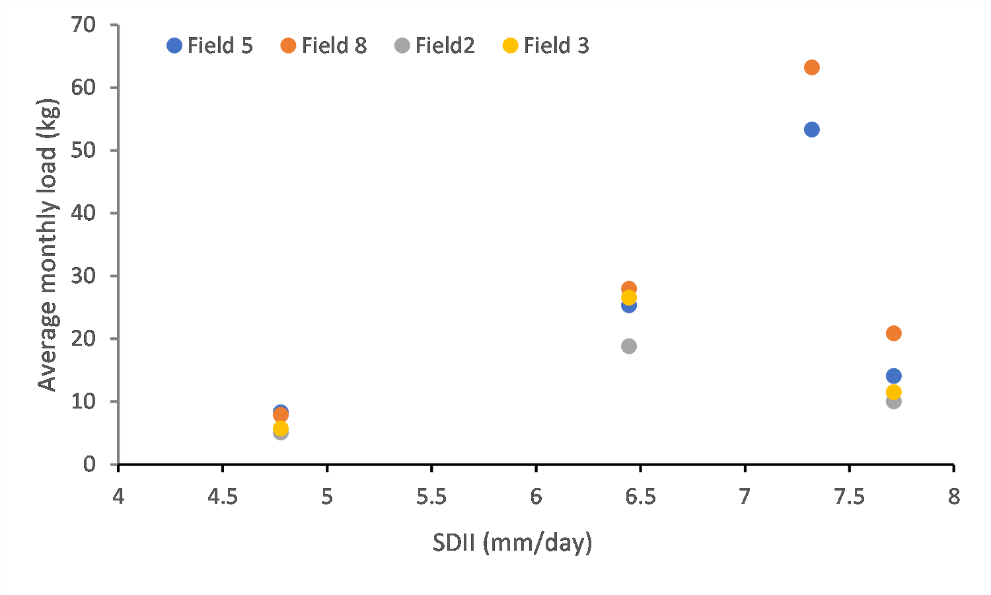
**

Figure A1: Average monthly sediment loads (2016-19) for field catchments 2 and 3 and 5 and 8 (2016-2020) on the NWFP plotted against the rainfall indices.


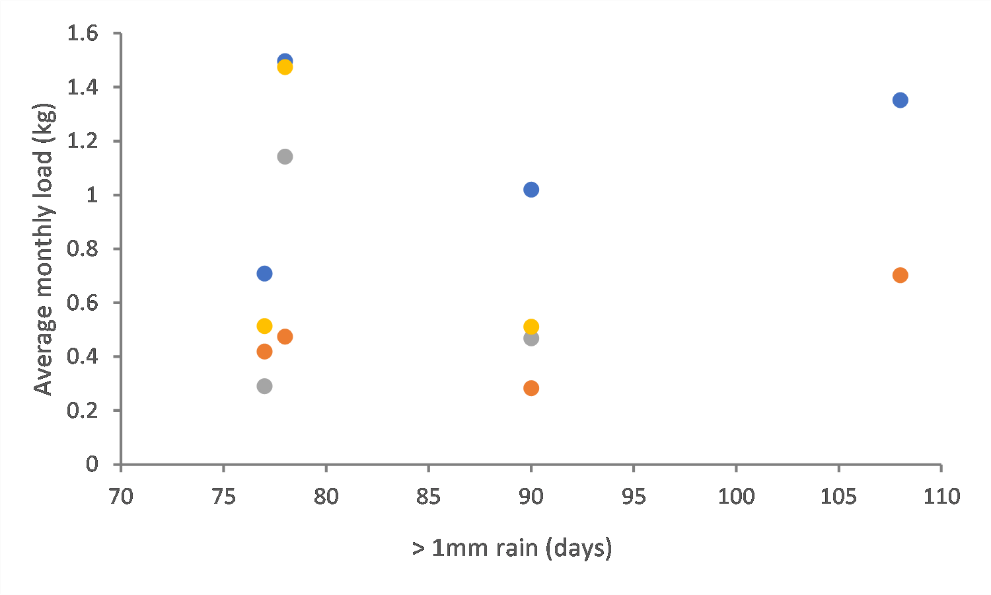


*
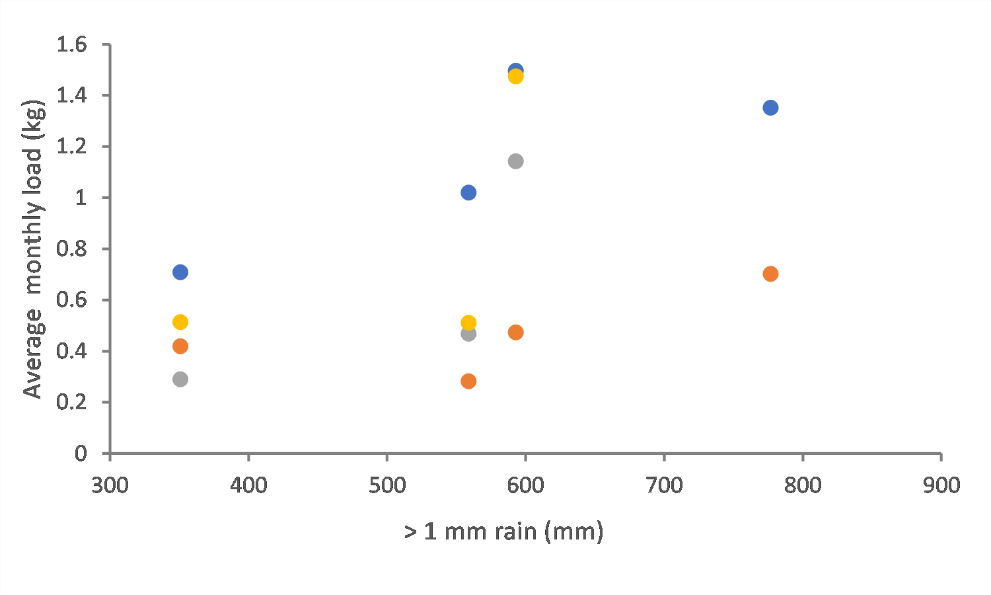
*


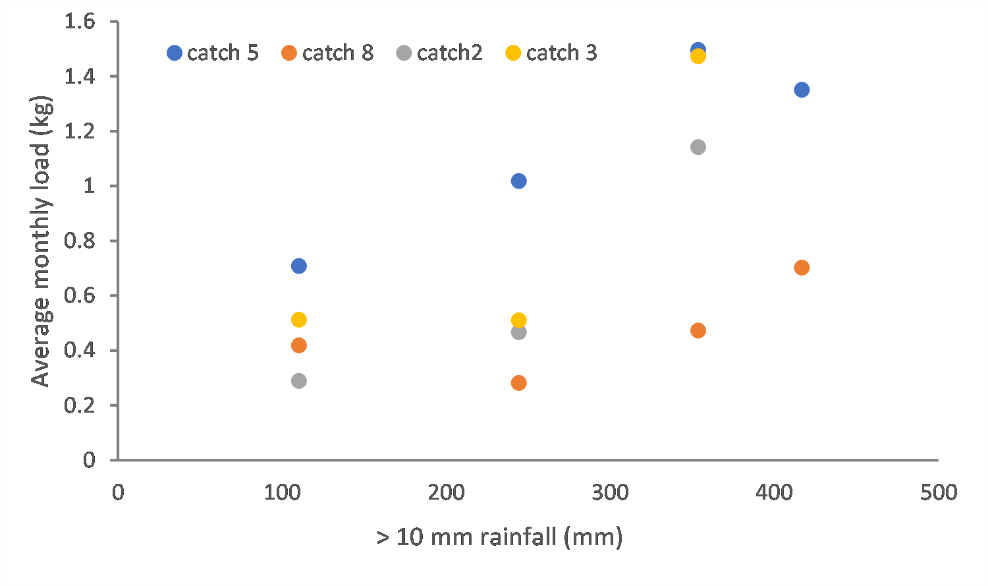


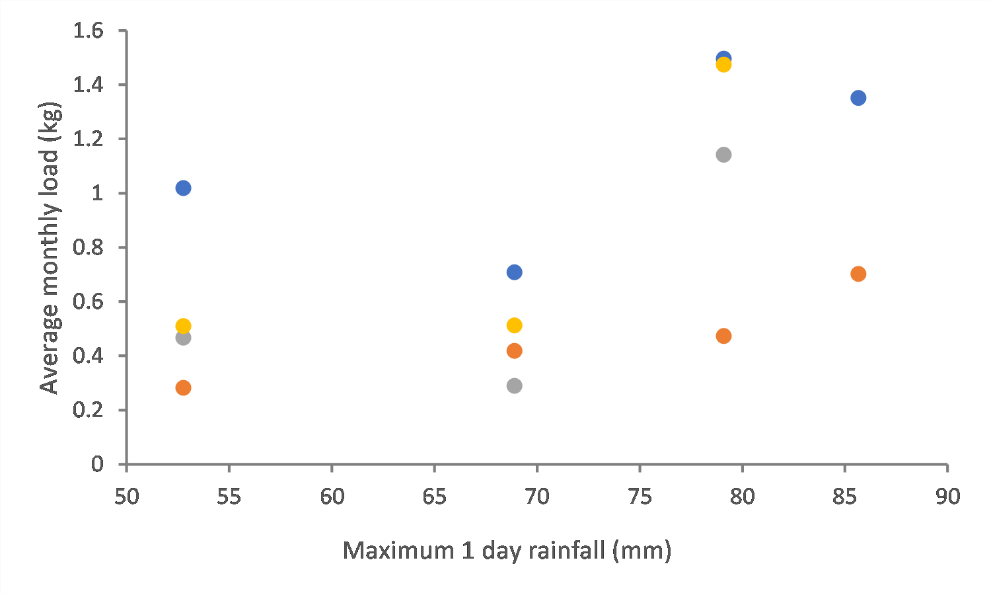


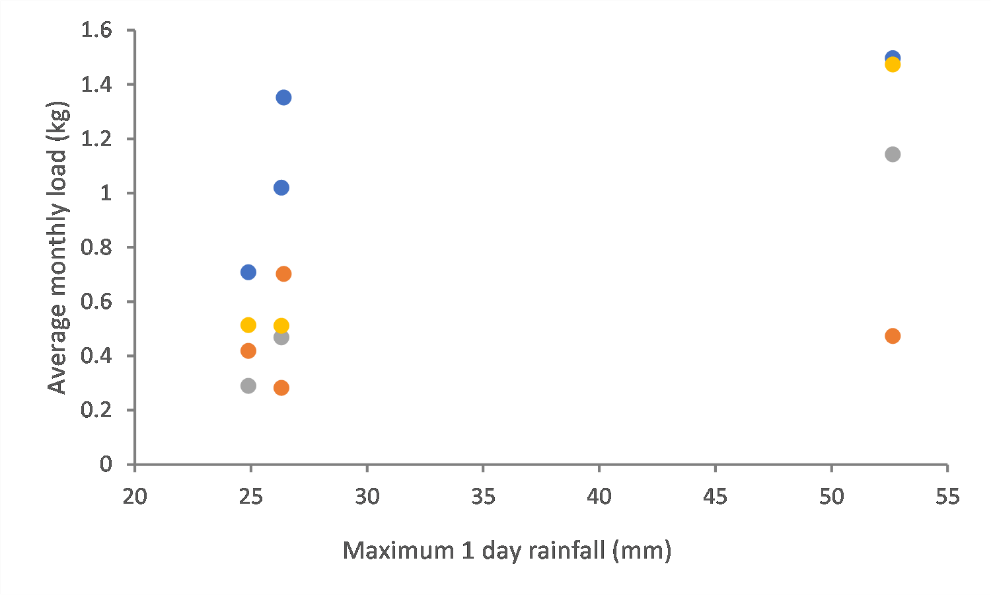


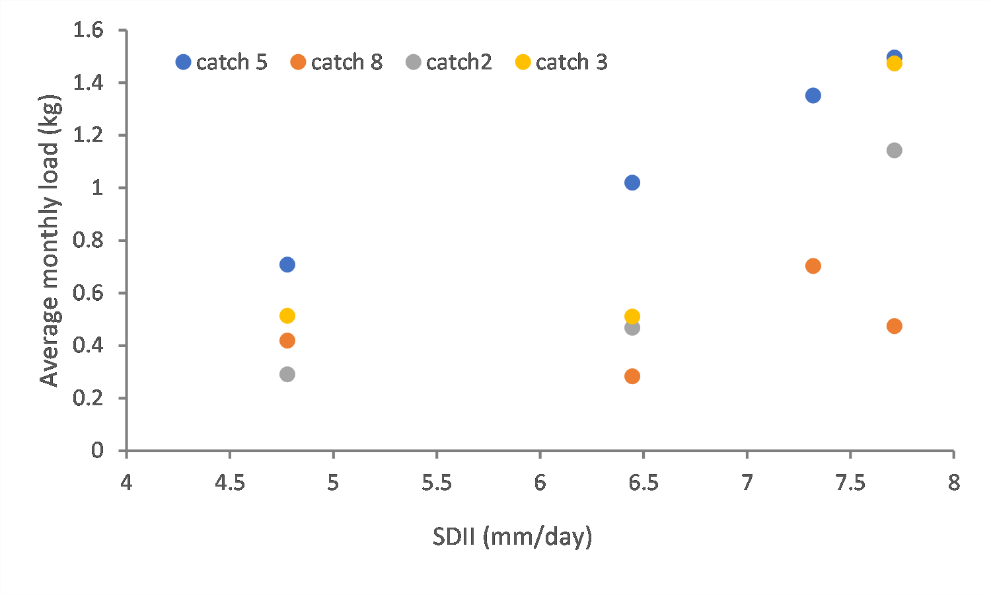


Figure A2: Average monthly nitrate loads (2016-19) for field catchments 2 and 3 and 5 and 8 (2016-2020) at field-scale on the NWFP plotted against the rainfall indices.

**Appendix B**

**Water quality load estimation algorithms and full results (for both the focus months of October-March and for April-September for comparison)**


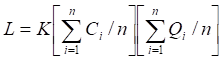
                     (Eq. B1)


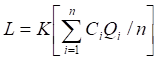
                                                 (Eq. B2)


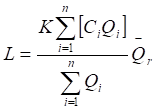
                                                  (Eq. B3)


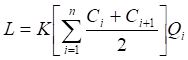
                                      (Eq. B4)


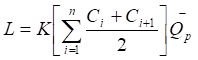
                                 (Eq. B5)


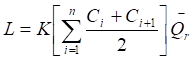
                                   (Eq. B6)


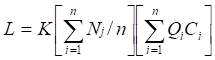
                                 (Eq. B7)

where:


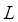
= load (kg or tonnes)


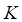
= a conversion factor to account for a) time period and b) units


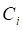
= concentration at sampling time
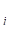
(mg l^-1^)


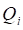
= discharge at sampling time
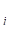
(m^3^ s^-1^)


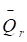
= mean flow for period of load estimate (derived from a continuous discharge record (m^3^ s^-1^)


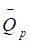
= mean flow over the period between water samples (derived from a continuous discharge record (m^3^ s^-1^)


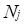
= number f time intervals in
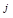
th stratum


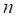
= number of water quality samples collected / number of days on which concentrations were determined


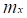
= mean daily flow of the days on which concentrations were determined


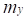
= mean daily loading for the days on which concentrations were determined


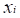
= individual measured flows


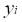
= daily loading for each day on which concentrations were determined

**Table B1. Estimated sediment loads (October-March) for the field catchments on the NWFP (kg ha^-1^ month^-1^)**

| **Field catchment** | **Month Year** | **1** | **2** | **3** | **4** | **5** | **6** | **7** |
| --- | --- | --- | --- | --- | --- | --- | --- | --- |
| Field 2 | Nov 16 | 12.5 | 23.8 | 8.9 | 11.1 | 11.3 | 11.1 | 11.5 |
|  | Dec 16 | 1.5 | 1.7 | 2.8 | 0.4 | 0.4 | 0.4 | 1.6 |
|  | Jan 17 | 3.2 | 5.2 | 4.6 | 2.5 | 2.6 | 2.5 | 2.7 |
|  | Feb 17 | 6.2 | 10.4 | 4.3 | 4.6 | 4.7 | 4.4 | 4.2 |
|  | Mar 17 | 5.0 | 8.7 | 4.9 | 5.2 | 5.3 | 5.2 | 5.4 |
|  | Oct 17 | 13.7 | 21.0 | 9.2 | 4.2 | 4.2 | 4.2 | 6.6 |
|  | Nov 17 | 18.2 | 31.9 | 10.0 | 12.4 | 12.4 | 12.4 | 12.7 |
|  | Dec 17 | 10.9 | 46.2 | 15.6 | 41.4 | 42.0 | 41.4 | 42.1 |
|  | Jan 18 | 13.2 | 33.0 | 8.2 | 27.7 | 28.8 | 27.7 | 28.3 |
|  | Feb 18 | 2.7 | 6.8 | 5.3 | 5.6 | 5.7 | 5.6 | 5.7 |
|  | Mar 18 | 13.2 | 19.3 | 5.0 | 19.2 | 19.3 | 19.2 | 19.2 |
|  | Oct 18 | 9.3 | 11.4 | 7.7 | 1.0 | 1.0 | 1.0 | 3.4 |
|  | Nov 18 | 8.9 | 17.9 | 6.0 | 14.3 | 14.4 | 14.3 | 14.4 |
|  | Dec 18 | 10.6 | 16.8 | 4.3 | 16.7 | 16.8 | 16.7 | 16.8 |
|  | Jan 19 | 2.7 | 5.3 | 4.4 | 4.0 | 4.0 | 4.0 | 4.0 |
|  | Feb 19 | 5.7 | 12.0 | 4.7 | 11.8 | 11.8 | 11.8 | 11.8 |
|  | Oct 19 | 9.5 | 30.0 | 73.6 | 27.7 | 28.1 | 27.7 | 28.0 |
|  | Nov 19 | 53.4 | 197.2 | 202.2 | 189.4 | 195.0 | 189.4 | 197.2 |
|  | Dec 19 | 137.5 | 726.7 | 577.6 | 594.4 | 621.2 | 602.7 | 612.1 |
|  | Jan 20 | 64.9 | 417.5 | 511.6 | 394.9 | 411.3 | 396.4 | 402.1 |
|  | Feb 20 | 184.1 | 883.4 | 533.2 | 799.3 | 843.5 | 799.3 | 870.3 |
|  | Mar 20 | 65.2 | 398.7 | 386.5 | 385.1 | 394.8 | 385.1 | 398.7 |
| Field 3 | Oct 16 | 2.2 | 2.3 | 10.6 | 0.0 | 0.0 | 0.0 | 2.3 |
|  | Nov 16 | 8.4 | 14.9 | 7.6 | 4.9 | 5.0 | 4.8 | 5.4 |
|  | Dec 16 | 0.9 | 1.1 | 2.4 | 0.6 | 0.6 | 0.6 | 0.6 |
|  | Jan 17 | 2.3 | 5.9 | 6.0 | 4.8 | 4.9 | 4.8 | 4.8 |
|  | Feb 17 | 4.4 | 13.2 | 8.3 | 8.8 | 8.9 | 8.8 | 8.7 |
|  | Mar 17 | 4.8 | 12.8 | 9.5 | 12.3 | 12.4 | 12.3 | 12.4 |
|  | Oct 17 | 2.9 | 8.7 | 10.0 | 4.6 | 4.7 | 4.7 | 4.8 |
|  | Nov 17 | 8.0 | 17.7 | 9.6 | 11.6 | 11.6 | 11.7 | 10.1 |
|  | Dec 17 | 15.6 | 86.6 | 29.6 | 75.2 | 76.5 | 75.2 | 76.8 |
|  | Jan 18 | 12.1 | 33.5 | 10.7 | 31.6 | 33.0 | 31.6 | 32.5 |
|  | Feb 18 | 3.2 | 9.9 | 7.9 | 9.0 | 9.2 | 8.9 | 9.2 |
|  | Mar 18 | 19.2 | 29.8 | 7.4 | 27.3 | 27.6 | 27.3 | 27.6 |
|  | Oct 18 | 1.9 | 2.9 | 4.8 | 0.7 | 0.7 | 0.7 | 1.3 |
|  | Nov 18 | 12.1 | 30.2 | 9.3 | 16.2 | 16.6 | 16.2 | 16.8 |
|  | Dec 18 | 9.8 | 17.4 | 4.9 | 17.3 | 17.3 | 17.3 | 17.4 |
|  | Jan 19 | 3.0 | 10.7 | 7.6 | 8.6 | 8.7 | 8.6 | 8.7 |
|  | Feb 19 | 4.7 | 15.7 | 7.1 | 14.0 | 14.1 | 14.0 | 14.2 |
|  | Oct 19 | 11.4 | 57.3 | 123.7 | 32.5 | 33.2 | 32.5 | 33.5 |
|  | Nov 19 | 105.2 | 539.7 | 424.1 | 502.1 | 518.7 | 502.5 | 501.5 |
|  | Dec 19 | 116.1 | 759.6 | 672.1 | 648.6 | 680.8 | 649.7 | 686.9 |
|  | Jan 20 | 44.6 | 368.8 | 496.0 | 336.1 | 350.7 | 337.0 | 357.9 |
|  | Feb 20 | 131.6 | 651.4 | 408.2 | 614.1 | 636.5 | 614.1 | 651.4 |
|  | Mar 20 | 49.3 | 264.0 | 256.6 | 247.1 | 250.5 | 247.4 | 250.4 |
| Field 5 | Oct 16 | 1.9 | 1.9 | 8.2 | 0.1 | 0.1 | 0.1 | 1.9 |
|  | Nov 16 | 18.6 | 33.8 | 15.5 | 7.5 | 7.7 | 7.4 | 9.5 |
|  | Dec 16 | 5.4 | 7.0 | 15.2 | 0.8 | 0.8 | 0.7 | 4.9 |
|  | Jan 17 | 3.6 | 8.9 | 12.4 | 4.9 | 5.1 | 4.9 | 4.8 |
|  | Feb 17 | 6.2 | 16.7 | 11.5 | 11.3 | 11.6 | 11.3 | 11.5 |
|  | Mar 17 | 6.7 | 18.5 | 14.4 | 17.1 | 17.8 | 17.1 | 17.8 |
|  | Oct 17 | 17.0 | 33.9 | 21.3 | 5.4 | 5.5 | 5.2 | 7.7 |
|  | Nov 17 | 7.8 | 14.0 | 9.8 | 9.0 | 9.1 | 9.4 | 6.1 |
|  | Dec 17 | 13.3 | 50.4 | 18.0 | 47.8 | 49.9 | 47.8 | 49.8 |
|  | Jan 18 | 16.8 | 46.6 | 13.0 | 43.2 | 45.9 | 43.2 | 45.2 |
|  | Feb 18 | 4.3 | 13.7 | 10.5 | 11.0 | 11.6 | 11.0 | 11.6 |
|  | Mar 18 | 20.4 | 33.8 | 8.7 | 33.0 | 33.8 | 33.0 | 33.8 |
|  | Oct 18 | 5.7 | 7.8 | 9.6 | 1.0 | 1.0 | 1.0 | 2.9 |
|  | Nov 18 | 13.5 | 26.5 | 8.2 | 18.0 | 18.4 | 18.0 | 18.1 |
|  | Dec 18 | 15.5 | 29.8 | 7.5 | 29.0 | 29.6 | 29.0 | 29.8 |
|  | Jan 19 | 6.1 | 11.1 | 7.4 | 6.4 | 6.7 | 6.4 | 6.6 |
|  | Feb 19 | 6.6 | 14.2 | 6.3 | 13.7 | 14.0 | 13.7 | 14.2 |
|  | Oct 19 | 9.1 | 20.4 | 51.2 | 12.2 | 12.5 | 12.2 | 12.2 |
|  | Nov 19 | 20.4 | 46.1 | 46.6 | 44.1 | 45.5 | 44.1 | 46.0 |
|  | Dec 19 | 30.2 | 71.1 | 56.4 | 67.4 | 69.7 | 67.6 | 68.3 |
|  | Jan 20 | 13.8 | 38.3 | 55.5 | 36.3 | 38.0 | 36.3 | 38.3 |
|  | Feb 20 | 41.1 | 94.6 | 54.3 | 90.4 | 93.6 | 90.4 | 94.6 |
|  | Mar 20 | 25.0 | 69.2 | 66.0 | 66.2 | 69.0 | 66.2 | 69.2 |
| Field 8 | Oct 16 | 3.2 | 3.5 | 4.6 | 0.3 | 0.3 | 0.3 | 2.0 |
|  | Nov 16 | 36.3 | 67.7 | 17.8 | 9.7 | 10.0 | 9.7 | 12.3 |
|  | Dec 16 | 1.3 | 1.8 | 6.1 | 0.1 | 0.1 | 0.1 | 1.8 |
|  | Jan 17 | 4.1 | 12.4 | 12.8 | 5.2 | 5.3 | 5.2 | 5.6 |
|  | Feb 17 | 9.8 | 26.9 | 10.6 | 13.2 | 13.4 | 13.1 | 13.6 |
|  | Mar 17 | 6.5 | 17.4 | 10.8 | 13.2 | 13.6 | 13.2 | 13.5 |
|  | Oct 17 | 21.0 | 43.6 | 18.8 | 7.4 | 7.5 | 7.4 | 9.1 |
|  | Nov 17 | 18.2 | 36.0 | 12.2 | 13.5 | 13.5 | 14.0 | 17.7 |
|  | Dec 17 | 17.2 | 80.7 | 26.5 | 66.4 | 69.2 | 66.4 | 69.3 |
|  | Jan 18 | 19.0 | 54.6 | 13.9 | 39.3 | 41.5 | 39.3 | 40.6 |
|  | Feb 18 | 4.2 | 19.8 | 14.0 | 11.1 | 11.6 | 11.1 | 11.5 |
|  | Mar 18 | 18.1 | 30.3 | 7.3 | 27.4 | 27.8 | 27.4 | 27.9 |
|  | Oct 18 | 4.6 | 5.2 | 7.6 | 0.2 | 0.2 | 0.2 | 3.1 |
|  | Nov 18 | 48.3 | 131.9 | 22.2 | 49.3 | 51.1 | 49.3 | 52.2 |
|  | Dec 18 | 15.3 | 29.9 | 6.7 | 29.2 | 29.8 | 29.2 | 29.9 |
|  | Jan 19 | 4.9 | 13.6 | 10.0 | 6.3 | 6.5 | 6.3 | 6.8 |
|  | Feb 19 | 6.8 | 21.6 | 8.8 | 16.1 | 16.4 | 16.1 | 16.4 |
|  | Oct 19 | 10.9 | 27.1 | 49.1 | 21.9 | 22.2 | 21.9 | 21.2 |
|  | Nov 19 | 15.5 | 33.6 | 31.7 | 59.6 | 60.0 | 49.1 | 18.9 |
|  | Dec 19 | 26.0 | 76.9 | 63.8 | 71.3 | 75.2 | 71.4 | 74.4 |
|  | Jan 20 | 11.9 | 56.0 | 84.3 | 51.9 | 55.8 | 51.9 | 56.0 |
|  | Feb 20 | 57.1 | 143.9 | 82.7 | 130.5 | 137.1 | 130.5 | 137.3 |
|  | Mar 20 | 18.8 | 68.4 | 64.3 | 66.0 | 68.0 | 66.0 | 68.3 |

**Table B2. Estimated nitrate loads (October-March) for the field catchments on the NWFP (kg ha^-1^ month^-1^)**

| **Field catchment** | **Month Year** | **1** | **2** | **3** | **4** | **5** | **6** | **7** |
| --- | --- | --- | --- | --- | --- | --- | --- | --- |
| Field 2 | Nov 16 | 1.34 | 0.96 | 0.36 | 0.47 | 0.47 | 0.48 | 0.50 |
|  | Dec 16 | 0.23 | 0.24 | 0.41 | 0.04 | 0.04 | 0.04 | 0.23 |
|  | Jan 17 | 0.42 | 0.37 | 0.38 | 0.20 | 0.20 | 0.20 | 0.24 |
|  | Feb 17 | 0.82 | 0.61 | 0.27 | 0.25 | 0.25 | 0.25 | 0.32 |
|  | Mar 17 | 0.51 | 0.36 | 0.22 | 0.24 | 0.24 | 0.24 | 0.22 |
|  | Oct 17 | 0.87 | 0.90 | 0.39 | 0.18 | 0.18 | 0.18 | 0.37 |
|  | Nov 17 | 1.21 | 1.03 | 0.32 | 0.41 | 0.41 | 0.41 | 0.46 |
|  | Dec 17 | 1.17 | 0.64 | 0.22 | 0.59 | 0.60 | 0.59 | 0.58 |
|  | Jan 18 | 1.32 | 0.68 | 0.17 | 0.64 | 0.64 | 0.64 | 0.58 |
|  | Feb 18 | 0.46 | 0.24 | 0.18 | 0.21 | 0.21 | 0.21 | 0.20 |
|  | Mar 18 | 1.09 | 0.59 | 0.15 | 0.59 | 0.59 | 0.59 | 0.58 |
|  | Oct 18 | 3.39 | 3.12 | 1.90 | 0.24 | 0.24 | 0.24 | 1.39 |
|  | Nov 18 | 3.00 | 2.39 | 0.80 | 1.94 | 1.93 | 1.93 | 1.91 |
|  | Dec 18 | 1.64 | 1.29 | 0.33 | 1.29 | 1.29 | 1.29 | 1.28 |
|  | Jan 19 | 0.68 | 0.55 | 0.46 | 0.44 | 0.44 | 0.44 | 0.41 |
|  | Feb 19 | 1.10 | 0.66 | 0.26 | 0.66 | 0.66 | 0.66 | 0.65 |
|  | Oct 19 | 3.66 | 3.37 | 11.61 | 4.85 | 4.84 | 5.05 | 2.89 |
|  | Nov 19 | 3.54 | 3.37 | 3.43 | 3.34 | 3.34 | 3.34 | 3.30 |
|  | Dec 19 | 2.82 | 1.47 | 1.17 | 1.66 | 1.67 | 1.61 | 1.23 |
|  | Jan 20 | 1.86 | 1.42 | 1.60 | 1.26 | 1.28 | 1.26 | 1.23 |
|  | Feb 20 | 3.07 | 2.03 | 1.03 | 1.63 | 1.68 | 1.63 | 1.63 |
|  | Mar 20 | 1.90 | 1.37 | 1.29 | 1.35 | 1.35 | 1.35 | 1.26 |
| Field 3 | Nov 16 | 1.66 | 1.53 | 0.74 | 0.49 | 0.49 | 0.49 | 0.67 |
|  | Dec 16 | 0.20 | 0.22 | 0.49 | 0.09 | 0.09 | 0.08 | 0.13 |
|  | Jan 17 | 0.79 | 0.79 | 0.80 | 0.66 | 0.66 | 0.66 | 0.64 |
|  | Feb 17 | 0.95 | 0.78 | 0.49 | 0.57 | 0.57 | 0.57 | 0.52 |
|  | Mar 17 | 0.71 | 0.54 | 0.41 | 0.54 | 0.54 | 0.54 | 0.52 |
|  | Oct 17 | 0.37 | 0.40 | 0.45 | 0.22 | 0.22 | 0.22 | 0.26 |
|  | Nov 17 | 0.73 | 0.61 | 0.33 | 0.43 | 0.43 | 0.43 | 0.34 |
|  | Dec 17 | 1.53 | 0.97 | 0.33 | 0.88 | 0.89 | 0.88 | 0.85 |
|  | Jan 18 | 1.30 | 0.67 | 0.21 | 0.68 | 0.68 | 0.68 | 0.64 |
|  | Feb 18 | 0.52 | 0.29 | 0.23 | 0.27 | 0.27 | 0.27 | 0.26 |
|  | Mar 18 | 1.05 | 0.58 | 0.15 | 0.54 | 0.55 | 0.54 | 0.53 |
|  | Oct 18 | 2.20 | 2.24 | 2.28 | 0.31 | 0.31 | 0.30 | 1.13 |
|  | Nov 18 | 5.69 | 3.91 | 1.20 | 2.22 | 2.22 | 2.22 | 2.38 |
|  | Dec 18 | 2.05 | 1.86 | 0.53 | 1.86 | 1.86 | 1.86 | 1.84 |
|  | Jan 19 | 1.37 | 1.33 | 0.96 | 1.12 | 1.12 | 1.12 | 1.08 |
|  | Feb 19 | 1.56 | 1.12 | 0.51 | 1.03 | 1.03 | 1.03 | 1.00 |
|  | Oct 19 | 11.43 | 9.91 | 3.15 | 10.10 | 10.02 | 10.10 | 9.91 |
|  | Nov 19 | 7.90 | 6.01 | 4.19 | 7.14 | 7.14 | 7.36 | 4.51 |
|  | Dec 19 | 3.98 | 2.27 | 2.07 | 2.58 | 2.59 | 2.55 | 1.98 |
|  | Jan 20 | 1.95 | 1.37 | 1.92 | 1.41 | 1.42 | 1.41 | 1.31 |
|  | Feb 20 | 2.71 | 2.03 | 1.33 | 2.12 | 2.12 | 2.13 | 1.87 |
|  | Mar 20 | 1.68 | 1.38 | 1.34 | 1.34 | 1.34 | 1.34 | 1.29 |
| Field 5 | Nov 16 | 3.02 | 1.95 | 0.90 | 0.47 | 0.47 | 0.47 | 0.89 |
|  | Dec 16 | 0.59 | 0.59 | 1.25 | 0.06 | 0.06 | 0.06 | 0.47 |
|  | Jan 17 | 0.92 | 0.79 | 1.15 | 0.51 | 0.51 | 0.51 | 0.45 |
|  | Feb 17 | 1.54 | 1.01 | 0.71 | 0.78 | 0.78 | 0.78 | 0.71 |
|  | Mar 17 | 1.23 | 0.90 | 0.71 | 0.90 | 0.90 | 0.90 | 0.88 |
|  | Oct 17 | 1.17 | 0.93 | 0.58 | 0.15 | 0.15 | 0.15 | 0.35 |
|  | Nov 17 |  |  |  | 1.31 | 1.31 | 1.46 |  |
|  | Dec 17 | 3.15 | 1.21 | 0.42 | 1.19 | 1.19 | 1.19 | 1.16 |
|  | Jan 18 | 3.09 | 1.46 | 0.41 | 1.48 | 1.48 | 1.48 | 1.40 |
|  | Feb 18 | 1.21 | 0.65 | 0.50 | 0.60 | 0.60 | 0.60 | 0.55 |
|  | Mar 18 | 2.71 | 1.17 | 0.30 | 1.17 | 1.17 | 1.17 | 1.16 |
|  | Oct 18 | 1.14 | 1.15 | 1.41 | 0.18 | 0.18 | 0.18 | 0.62 |
|  | Nov 18 | 5.39 | 3.31 | 1.03 | 2.35 | 2.35 | 2.35 | 2.24 |
|  | Dec 18 | 3.44 | 2.26 | 0.57 | 2.26 | 2.26 | 2.26 | 2.24 |
|  | Jan 19 | 1.79 | 1.26 | 0.84 | 0.78 | 0.78 | 0.78 | 0.88 |
|  | Feb 19 | 2.70 | 1.40 | 0.62 | 1.40 | 1.40 | 1.40 | 1.38 |
|  | Nov 19 | 1.22 | 1.03 | 2.59 | 0.65 | 0.65 | 0.65 | 0.61 |
|  | Dec 19 | 2.75 | 2.01 | 2.03 | 2.01 | 2.01 | 2.01 | 1.98 |
|  | Jan 20 | 2.76 | 1.47 | 1.16 | 1.58 | 1.59 | 1.57 | 1.39 |
|  | Feb 20 | 2.04 | 1.17 | 1.25 | 1.17 | 1.17 | 1.17 | 1.16 |
| Field 8 | Nov 16 | 2.70 | 1.92 | 0.51 | 0.29 | 0.29 | 0.30 | 0.55 |
|  | Dec 16 | 0.16 | 0.16 | 0.49 | 0.02 | 0.02 | 0.02 | 0.16 |
|  | Jan 17 | 0.93 | 0.78 | 1.09 | 0.49 | 0.49 | 0.49 | 0.45 |
|  | Feb 17 | 1.74 | 1.06 | 0.43 | 0.58 | 0.57 | 0.58 | 0.63 |
|  | Mar 17 | 0.65 | 0.43 | 0.27 | 0.36 | 0.36 | 0.36 | 0.34 |
|  | Oct 17 | 0.75 | 0.62 | 0.26 | 0.12 | 0.12 | 0.12 | 0.17 |
|  | Nov 17 | 0.82 | 0.47 | 0.16 | 0.21 | 0.21 | 0.21 | 0.28 |
|  | Dec 17 | 1.06 | 0.53 | 0.17 | 0.46 | 0.46 | 0.46 | 0.45 |
|  | Jan 18 | 0.84 | 0.44 | 0.11 | 0.36 | 0.36 | 0.36 | 0.32 |
|  | Feb 18 | 0.38 | 0.15 | 0.11 | 0.10 | 0.11 | 0.10 | 0.09 |
|  | Mar 18 | 0.85 | 0.42 | 0.10 | 0.38 | 0.38 | 0.38 | 0.38 |
|  | Oct 18 | 0.53 | 0.51 | 0.74 | 0.02 | 0.02 | 0.02 | 0.38 |
|  | Nov 18 | 2.84 | 1.71 | 0.30 | 0.70 | 0.71 | 0.70 | 0.81 |
|  | Dec 18 | 1.07 | 0.83 | 0.19 | 0.82 | 0.83 | 0.82 | 0.82 |
|  | Jan 19 | 0.41 | 0.31 | 0.23 | 0.17 | 0.17 | 0.17 | 0.19 |
|  | Feb 19 | 0.52 | 0.34 | 0.14 | 0.26 | 0.26 | 0.26 | 0.26 |
|  | Oct 19 | 0.75 | 0.93 | 1.73 | 0.75 | 0.75 | 0.75 | 0.71 |
|  | Nov 19 | 0.94 | 0.87 | 0.88 | 0.97 | 0.97 | 1.00 | 0.47 |
|  | Dec 19 | 1.00 | 0.75 | 0.62 | 0.78 | 0.78 | 0.78 | 0.71 |
|  | Jan 20 | 0.57 | 0.44 | 0.65 | 0.43 | 0.43 | 0.43 | 0.43 |
|  | Feb 20 | 1.08 | 0.62 | 0.35 | 0.60 | 0.60 | 0.60 | 0.58 |

**Table B3. Estimated sediment loads (April-September) for the field catchments on the NWFP (kg ha^-1^ month^-1^)***

| **Month Year** | **Field 2** | **Field 3** | **Field 5** | **Field 8** |
| --- | --- | --- | --- | --- |
| Apr 17 | 1.1 | 1.1 | 1.6 | 1.5 |
| May 17 | **1.8** | 3.5 | 5.3 | 8.1 |
| Jun 17 | **0.2** | **2.0** | **4.0** | **2.1** |
| Jul 17 | **1.3** | 2.6 | **5.8** | **5.9** |
| Aug 17 | 0.2 | 0.7 | 3.5 | 2.5 |
| Sep 17 | 3.7 | 6.3 | 4.9 | 9.2 |
| Apr 18 | 10.6 | 17.8 | 18.6 | 18.6 |
| May 18 | **9.2** | 0.5 | **4.5** | 1.1 |
| Jun 18 | **0.4** | **0.0** | **0.1** | **0.9** |
| Jul 18 | **0.0** | **1.1** | **0.0** | 0.0 |
| Aug 18 | **2.1** | 0.8 | **7.5** | **2.0** |
| Sep 18 | **1.7** | **1.6** | **3.0** | **2.9** |

* For these values in red, only limited samples were collected when flow rates exceeded 0.2 l s^-1^ and high uncertainty and over-estimation are expected.

**Table B4. Estimated nitrate loads (April-September) for the field catchments on the NWFP (kg ha^-1^ month^-1^)***

| **Month Year** | **Field 2** | **Field 3** | **Field 5** | **Field 8** |
| --- | --- | --- | --- | --- |
| Apr 17 | 0.1 | 0.2 | 0.3 | 0.1 |
| May 17 | **0.1** | 0.2 | 0.3 | 0.2 |
| Jun 17 | **0.0** | **0.2** | **0.5** | **0.2** |
| Jul 17 | **0.1** | 0.4 | 0.7 | 0.3 |
| Aug 17 | **0.0** | 0.1 | **0.4** | **0.1** |
| Sep 17 | 0.3 | 0.3 | 0.3 | 0.2 |
| Apr 18 | **0.3** | 0.3 | 0.8 | 0.3 |
| May 18 | **0.4** | 0.1 | **0.3** | **0.1** |
| Jun 18 | **0.0** | **0.0** | **0.0** | **0.0** |
| Jul 18 | **0.0** | **0.3** | **0.0** | **0.0** |
| Aug 18 | **1.2** | **0.8** | **1.9** | **0.4** |
| Sep 18 | **0.9** | **0.7** | **1.3** | **0.5** |

* For these values in red, only limited samples were collected when flow rates exceeded 0.2 l s^-1^ and high uncertainty and over-estimation are expected.

**Table B5. Estimated flows (April-September) for the field catchments on the NWFP (l s^-1^)**

|  | **Field 2** | | |  | **Field 3** | | | | **Field 5** | | | | **Field 8** | | | |
| --- | --- | --- | --- | --- | --- | --- | --- | --- | --- | --- | --- | --- | --- | --- | --- | --- |
| **Month Year** | **Mean** | **Median** | **P95*** | **IQR^+^** | **Mean** | **Median** | **P95*** | **IQR^+^** | **Mean** | **Median** | **P95*** | **IQR^+^** | **Mean** | **Median** | **P95*** | **IQR^+^** |
| Apr 17 | 0.05 | 0.01 | 0.14 | 0.00 | 0.08 | 0.03 | 0.33 | 0.05 | 0.13 | 0.09 | 0.36 | 0.05 | 0.07 | 0.02 | 0.20 | 0.02 |
| May 17 | 0.03 | 0.01 | 0.05 | 0.01 | 0.07 | 0.03 | 0.23 | 0.02 | 0.07 | 0.03 | 0.15 | 0.02 | 0.06 | 0.00 | 0.07 | 0.00 |
| Jun 17 | 0.02 | 0.01 | 0.03 | 0.01 | 0.03 | 0.02 | 0.10 | 0.01 | 0.01 | 0.00 | 0.05 | 0.00 | 0.01 | 0.00 | 0.03 | 0.00 |
| Jul 17 | 0.01 | 0.01 | 0.02 | 0.01 | 0.08 | 0.02 | 0.33 | 0.01 | 0.08 | 0.00 | 0.12 | 0.00 | 0.18 | 0.00 | 0.19 | 0.03 |
| Aug 17 | 0.02 | 0.01 | 0.04 | 0.02 | 0.03 | 0.02 | 0.10 | 0.02 | 0.01 | 0.00 | 0.02 | 0.00 | 0.00 | 0.00 | 0.05 | 0.00 |
| Sep 17 | 0.19 | 0.06 | 0.43 | 0.08 | 0.34 | 0.14 | 0.90 | 0.14 | 0.14 | 0.03 | 0.43 | 0.06 | 0.30 | 0.03 | 0.90 | 0.07 |
| Apr 18 | 1.03 | 0.20 | 4.01 | 0.29 | 1.18 | 0.23 | 4.51 | 0.43 | 1.26 | 0.40 | 4.11 | 0.33 | 1.18 | 0.23 | 4.61 | 0.23 |
| May 18 | 0.03 | 0.03 | 0.03 | 0.01 | 0.05 | 0.03 | 0.12 | 0.05 | 0.05 | 0.05 | 0.09 | 0.01 | 0.22 | 0.05 | 0.73 | 0.41 |
| Jun 18 | 0.01 | 0.01 | 0.02 | 0.00 | 0.01 | 0.01 | 0.02 | 0.00 | 0.00 | 0.00 | 0.01 | 0.01 | 0.01 | 0.00 | 0.00 | 0.00 |
| Jul 18 | 0.00 | 0.00 | 0.01 | 0.00 | 0.00 | 0.00 | 0.02 | 0.00 | 0.00 | 0.00 | 0.00 | 0.00 | 0.00 | 0.00 | 0.00 | 0.00 |
| Aug 18 | 0.01 | 0.00 | 0.03 | 0.01 | 0.05 | 0.01 | 0.18 | 0.09 | 0.00 | 0.00 | 0.00 | 0.00 | 0.00 | 0.00 | 0.00 | 0.00 |
| Sep 18 | 0.01 | 0.00 | 0.02 | 0.00 | 0.01 | 0.00 | 0.02 | 0.00 | 0.01 | 0.00 | 0.01 | 0.00 | 0.01 | 0.00 | 0.02 | 0.00 |

* P95: 95^th^ percentile; ^+^ IQR: Inter-Quartile Range

**Table B6. Estimated sediment loads (October-March) for the catchments in the UTRO (kg ha^-1^ month^-1^)**

| **Catchment** | **Month Year** | **1** | **2** | **3** | **4** | **5** | **6** | **7** |
| --- | --- | --- | --- | --- | --- | --- | --- | --- |
| Pecketsford | Oct 18 | 6.4 | 14.8 | 28.1 | 14.5 | 14.6 | 14.5 | 13.4 |
|  | Nov 18 | 22.7 | 58.5 | 47.2 | 52.2 | 52.6 | 52.2 | 51.4 |
|  | Dec 18 | 32.7 | 41.8 | 19.7 | 32.8 | 32.9 | 33.6 | 27.6 |
|  | Jan 19 | 6.6 | 9.6 | 18.1 | 8.6 | 8.6 | 8.4 | 7.1 |
|  | Feb 19 | 16.1 | 30.4 | 25.4 | 28.2 | 28.3 | 28.2 | 27.8 |
|  | Mar 19 | 18.0 | 33.0 | 28.0 | 31.2 | 31.5 | 31.2 | 30.8 |
|  | Oct 19 | 21.2 | 37.2 | 31.7 | 34.1 | 34.3 | 34.2 | 33.4 |
|  | Nov 19 | 39.6 | 69.5 | 44.4 | 58.0 | 58.3 | 58.2 | 52.4 |
|  | Dec 19 | 64.8 | 113.6 | 58.3 | 100.1 | 100.6 | 100.9 | 85.8 |
|  | Jan 20 | 35.9 | 66.7 | 48.4 | 62.4 | 62.9 | 62.5 | 61.5 |
|  | Feb 20 | 89.7 | 221.1 | 97.0 | 201.3 | 203.1 | 201.4 | 197.4 |
|  | Mar 20 | 54.7 | 88.4 | 43.5 | 87.4 | 87.9 | 87.4 | 87.9 |
| Upper | Oct 18 | 0.0 | 0.0 | 0.1 | 0.0 | 0.0 | 0.0 | 0.0 |
| Ratcombe | Nov 18 | 12.7 | 23.4 | 17.4 | 32.2 | 32.4 | 32.5 | 13.2 |
|  | Dec 18 | 9.8 | 11.2 | 4.7 | 9.2 | 9.2 | 9.1 | 7.3 |
|  | Jan 19 | 2.4 | 3.1 | 4.8 | 2.7 | 2.7 | 2.7 | 2.2 |
|  | Feb 19 | 5.4 | 8.3 | 6.7 | 7.5 | 7.5 | 7.5 | 7.3 |
|  | Mar 19 | 5.0 | 7.2 | 6.6 | 7.1 | 7.2 | 7.2 | 6.7 |
|  | Oct 19 | 7.5 | 9.1 | 11.0 | 8.0 | 8.0 | 8.2 | 6.4 |
|  | Nov 19 | 11.2 | 15.9 | 9.2 | 14.0 | 14.0 | 13.7 | 11.9 |
|  | Dec 19 | 13.8 | 21.4 | 9.6 | 18.6 | 18.6 | 18.5 | 18.2 |
|  | Jan 20 | 12.6 | 16.8 | 8.0 | 12.8 | 12.8 | 12.8 | 10.8 |
|  | Feb 20 | 16.3 | 29.9 | 13.0 | 27.7 | 27.8 | 27.7 | 25.4 |
|  | Mar 20 | 5.3 | 6.1 | 3.8 | 6.1 | 6.1 | 6.1 | 6.1 |
| Lower | Oct 18 | 1.5 | 1.5 | 9.1 | 1.3 | 1.3 | 1.3 | 1.3 |
| Ratcombe | Nov 18 | 36.6 | 90.5 | 76.2 | 90.4 | 91.0 | 90.8 | 67.1 |
|  | Dec 18 | 46.9 | 56.1 | 25.3 | 45.0 | 45.1 | 46.6 | 37.1 |
|  | Jan 19 | 8.2 | 11.4 | 20.6 | 10.1 | 10.1 | 9.8 | 8.2 |
|  | Feb 19 | 21.0 | 37.1 | 28.0 | 34.4 | 34.6 | 34.4 | 33.9 |
|  | Mar 19 | 25.4 | 44.6 | 36.9 | 42.2 | 42.4 | 42.2 | 41.8 |
|  | Oct 19 | 27.6 | 48.6 | 50.7 | 42.8 | 42.9 | 42.7 | 42.0 |
|  | Nov 19 | 66.6 | 122.8 | 58.9 | 141.3 | 141.3 | 145.9 | 75.0 |
|  | Dec 19 | 77.7 | 146.2 | 63.7 | 127.4 | 127.8 | 127.6 | 121.2 |
|  | Jan 20 | 37.8 | 77.7 | 58.9 | 70.3 | 70.8 | 70.3 | 69.9 |
|  | Feb 20 | 186.7 | 568.8 | 227.4 | 553.7 | 554.6 | 564.0 | 403.8 |
|  | Mar 20 | 52.7 | 119.3 | 69.2 | 122.4 | 123.8 | 122.2 | 119.2 |

**Table B7. Estimated nitrate loads (October-March) for the catchments in the UTRO (kg ha^-1^ month^-1^)**

| **Catchment** | **Month Year** | **1** | **2** | **3** | **4** | **5** | **6** | **7** |
| --- | --- | --- | --- | --- | --- | --- | --- | --- |
| Pecketsford | Oct 18 | 1.50 | 2.73 | 5.17 | 2.61 | 2.61 | 2.61 | 2.48 |
|  | Nov 18 | 4.44 | 5.93 | 4.73 | 5.41 | 5.41 | 5.41 | 5.33 |
|  | Dec 18 | 17.28 | 17.11 | 8.06 | 13.09 | 13.09 | 14.33 | 11.73 |
|  | Jan 19 |  |  |  | 1.95 | 1.95 | 2.09 |  |
|  | Feb 19 | 0.81 | 0.79 | 1.84 | 3.66 | 3.66 | 2.12 | 0.37 |
|  | Mar 19 | 3.53 | 4.14 | 3.51 | 4.05 | 4.05 | 4.06 | 3.87 |
|  | Oct 19 | 3.02 | 2.94 | 2.51 | 2.78 | 2.78 | 2.79 | 2.65 |
|  | Nov 19 | 1.47 | 1.57 | 1.02 | 1.67 | 1.67 | 1.70 | 1.32 |
|  | Dec 19 |  |  |  | 2.66 | 2.66 | 2.71 |  |
|  | Jan 20 | 1.20 | 1.22 | 1.34 | 1.90 | 1.90 | 1.81 | 1.13 |
|  | Feb 20 | 1.94 | 1.93 | 1.37 | 2.58 | 2.58 | 2.55 | 1.18 |
|  | Mar 20 | 3.63 | 3.65 | 1.80 | 3.65 | 3.65 | 3.65 | 3.65 |
| Upper | Oct 18 | 0.28 | 0.28 | 0.82 | 0.28 | 0.28 | 0.28 | 0.28 |
| Ratcombe | Nov 18 | 2.04 | 2.21 | 1.77 | 2.09 | 2.09 | 2.09 | 1.99 |
|  | Dec 18 | 4.65 | 4.65 | 1.94 | 4.08 | 4.08 | 4.23 | 3.05 |
|  | Jan 19 | 0.82 | 0.85 | 1.32 | 0.90 | 0.90 | 0.86 | 0.61 |
|  | Feb 19 | 1.35 | 1.47 | 1.18 | 1.36 | 1.36 | 1.36 | 1.30 |
|  | Mar 19 | 1.05 | 1.16 | 1.06 | 1.14 | 1.14 | 1.14 | 1.08 |
|  | Oct 19 | 0.95 | 0.95 | 1.15 | 0.92 | 0.92 | 0.92 | 0.82 |
|  | Nov 19 | 1.72 | 1.72 | 1.02 | 1.78 | 1.78 | 1.80 | 1.51 |
|  | Dec 19 | 3.35 | 3.44 | 1.54 | 3.18 | 3.18 | 3.17 | 2.92 |
|  | Jan 20 | 2.16 | 2.20 | 1.30 | 2.29 | 2.29 | 2.29 | 2.04 |
|  | Feb 20 | 2.59 | 2.62 | 1.14 | 2.92 | 2.92 | 2.93 | 2.23 |
|  | Mar 20 | 1.60 | 1.61 | 1.00 | 1.61 | 1.61 | 1.61 | 1.61 |
| Lower | Oct 18 | 0.21 | 0.21 | 1.45 | 0.21 | 0.21 | 0.21 | 0.21 |
| Ratcombe | Nov 18 | 4.28 | 4.54 | 3.48 | 4.17 | 4.17 | 4.17 | 4.10 |
|  | Dec 18 | 5.98 | 5.90 | 2.66 | 5.18 | 5.18 | 5.26 | 3.97 |
|  | Jan 19 |  |  |  | 1.13 | 1.13 | 1.11 |  |
|  | Feb 19 | 2.90 | 2.91 | 1.67 | 2.09 | 2.09 | 2.09 | 1.92 |
|  | Mar 19 | 2.82 | 3.16 | 2.62 | 3.15 | 3.15 | 3.15 | 2.96 |
|  | Oct 19 | 2.21 | 2.23 | 2.32 | 2.10 | 2.10 | 2.10 | 1.93 |
|  | Nov 19 | 3.57 | 3.54 | 1.75 | 3.59 | 3.59 | 3.61 | 3.00 |
|  | Dec 19 | 7.12 | 7.23 | 3.16 | 6.59 | 6.59 | 6.58 | 6.36 |
|  | Jan 20 | 3.41 | 3.32 | 2.52 | 3.17 | 3.17 | 3.17 | 2.98 |
|  | Feb 20 | 5.73 | 5.35 | 2.12 | 5.33 | 5.33 | 5.38 | 3.80 |
|  | Mar 20 | 6.56 | 6.49 | 3.75 | 6.49 | 6.49 | 6.49 | 6.49 |

**Table B8. Estimated sediment loads (April-September) for the catchments in the UTRO (kg ha^-1^ month^-1^)**

| **Month Year** | **Upper Ratcombe** | **Lower Ratcombe** | **Pecketsford** |
| --- | --- | --- | --- |
| Apr 19 | 1.1 | 5.0 | 3.6 |
| May 19 | 2.4 | 8.8 | 3.7 |
| Jun 19 | 1.8 | 3.8 | 4.3 |
| Jul 19 | 2.4 | 5.1 | 1.7 |
| Aug 19 | 0.6 | 1.5 | 5.6 |
| Sep 19 | 1.0 | 5.6 | 7.1 |

**Table B9. Estimated nitrate loads (April-September) for the catchments in the UTRO (kg ha^-1^ month^-1^)**

| **Month Year** | **Upper Ratcombe** | **Lower Ratcombe** | **Pecketsford** |
| --- | --- | --- | --- |
| Apr 19 | 0.22 | 0.56 | 0.57 |
| May 19 | 0.10 | 0.19 | 0.23 |
| Jun 19 | 0.09 | 0.20 | 0.58 |
| Jul 19 | 0.02 | 0.05 | 0.21 |
| Aug 19 | 0.07 | 0.10 | 0.64 |
| Sep 19 | 0.13 | 0.37 | 1.08 |

**Table B10. Estimated flows (October-March) for the catchments in the URTO (l s^-1^)**

| **Catchment** | **Month Year** | **Mean** | **Median** | **P95*** | **IQR^+^** | **Samples** |
| --- | --- | --- | --- | --- | --- | --- |
| Upper | Oct 18 | 12.6 | 12.3 | 16.6 | 5.6 | 672 |
| Ratcombe | Nov 18 | 46.7 | 38.4 | 103.3 | 31.6 | 2880 |
|  | Dec 18 | 79.6 | 77.7 | 125.2 | 38.9 | 2976 |
|  | Jan 19 | 24.7 | 23.5 | 45.9 | 9.6 | 2976 |
|  | Feb 19 | 42.3 | 40.6 | 90.9 | 33.2 | 2688 |
|  | Mar 19 | 39.1 | 34.1 | 80.4 | 33.1 | 2976 |
|  | Oct 19 | 30.3 | 26.7 | 59.3 | 16.7 | 2976 |
|  | Nov 19 | 61.4 | 55.2 | 107.6 | 16.2 | 2880 |
|  | Dec 19 | 75.2 | 55.1 | 210.9 | 29.6 | 2976 |
|  | Jan 20 | 62.9 | 44.4 | 152.6 | 39.8 | 2976 |
|  | Feb 20 | 91.2 | 78.0 | 205.0 | 54.4 | 2784 |
|  | Mar 20 | 46.9 | 42.3 | 96.3 | 31.7 | 2976 |
| Lower | Oct 18 | 21.5 | 19.9 | 25.0 | 3.8 | 612 |
| Ratcombe | Nov 18 | 177.2 | 115.6 | 545.6 | 175.0 | 2880 |
|  | Dec 18 | 286.7 | 291.9 | 493.3 | 146.9 | 2976 |
|  | Jan 19 | 77.8 | 59.5 | 162.7 | 48.7 | 2976 |
|  | Feb 19 | 172.7 | 120.4 | 450.9 | 176.0 | 2688 |
|  | Mar 19 | 163.3 | 127.1 | 409.3 | 158.6 | 2976 |
|  | Oct 19 | 131.4 | 87.2 | 317.0 | 117.0 | 2976 |
|  | Nov 19 | 281.5 | 238.0 | 559.4 | 106.8 | 2880 |
|  | Dec 19 | 294.6 | 228.3 | 712.5 | 192.7 | 2976 |
|  | Jan 20 | 176.9 | 123.2 | 422.0 | 80.6 | 2976 |
|  | Feb 20 | 363.6 | 240.1 | 1142.8 | 296.1 | 2784 |
|  | Mar 20 | 169.7 | 154.5 | 425.0 | 169.0 | 2976 |
| Pecketsford | Oct 18 | 1039.7 | 708.0 | 3221.3 | 501.5 | 2152 |
|  | Nov 18 | 2457.1 | 1568.0 | 7799.9 | 1809.0 | 2880 |
|  | Dec 18 | 3812.1 | 3439.5 | 7467.0 | 2127.7 | 2976 |
|  | Jan 19 | 1086.3 | 968.5 | 1957.3 | 484.3 | 2976 |
|  | Feb 19 | 2264.4 | 1553.5 | 5738.1 | 1809.9 | 2688 |
|  | Mar 19 | 2285.9 | 1760.1 | 6101.2 | 1584.2 | 2976 |
|  | Oct 19 | 2320.1 | 1793.8 | 5191.6 | 1088.1 | 2976 |
|  | Nov 19 | 3091.3 | 2674.4 | 5829.9 | 1298.5 | 2880 |
|  | Dec 19 | 3739.6 | 2928.1 | 8812.8 | 2482.2 | 2976 |
|  | Jan 20 | 2713.6 | 1953.9 | 6656.1 | 1077.8 | 2976 |
|  | Feb 20 | 4858.3 | 3334.3 | 17239.1 | 3247.1 | 2784 |
|  | Mar 20 | 3061.9 | 2952.7 | 6395.0 | 2460.8 | 2976 |

* P95: 95^th^ percentile; ^+^ IQR: Inter-Quartile Range

**Table B11. Estimated flows (April-September) for the catchments in the URTO (l s^-1^)**

| **Catchment** | **Month Year** | **Mean** | **Median** | **P95*** | **IQR^+^** | **Samples** |
| --- | --- | --- | --- | --- | --- | --- |
| Upper | Apr-19 | 12.0 | 12.5 | 18.8 | 5.7 | 2852 |
| Ratcombe | May-19 | 5.4 | 3.7 | 11.2 | 5.4 | 2970 |
|  | Jun-19 | 5.5 | 4.8 | 12.3 | 4.1 | 1943 |
|  | Jul-19 | 1.6 | 0.1 | 10.4 | 0.1 | 1929 |
|  | Aug-19 | 4.2 | 3.2 | 10.9 | 3.1 | 2971 |
|  | Sep-19 | 6.8 | 2.8 | 33.4 | 3.9 | 2821 |
| Lower | Apr-19 | 46.1 | 42.1 | 83.7 | 19.9 | 2880 |
| Ratcombe | May-19 | 24.3 | 19.9 | 43.5 | 9.4 | 2882 |
|  | Jun-19 | 22.6 | 17.6 | 49.3 | 13.9 | 2880 |
|  | Jul-19 | 7.9 | 5.4 | 21.9 | 3.4 | 2922 |
|  | Aug-19 | 13.3 | 10.8 | 31.8 | 6.9 | 2895 |
|  | Sep-19 | 21.4 | 6.1 | 112.3 | 7.9 | 2854 |
| Pecketsford | Apr-19 | 717.1 | 636.4 | 1286.3 | 286.4 | 2880 |
|  | May-19 | 365.5 | 317.3 | 630.2 | 94.5 | 2926 |
|  | Jun-19 | 627.3 | 498.6 | 1509.7 | 359.1 | 2880 |
|  | Jul-19 | 320.3 | 268.1 | 592.7 | 85.9 | 2976 |
|  | Aug-19 | 634.8 | 492.7 | 1566.7 | 227.1 | 2975 |
|  | Sep-19 | 887.0 | 401.6 | 3180.2 | 497.8 | 2879 |

* P95: 95^th^ percentile; ^+^ IQR: Inter-Quartile Range

**Figure B1: Comparison of rainfall for all months (2016-2020) with the climatic baseline (1981-2010).**


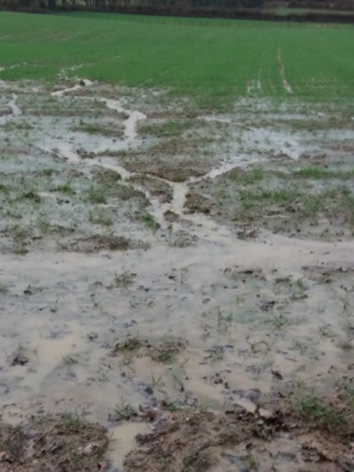

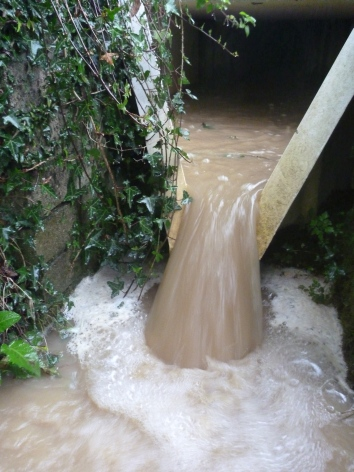


Picture A: Rilling and surface runoff from arable land Picture B: Turbid runoff from arable land

on the NWFP on the NWFP


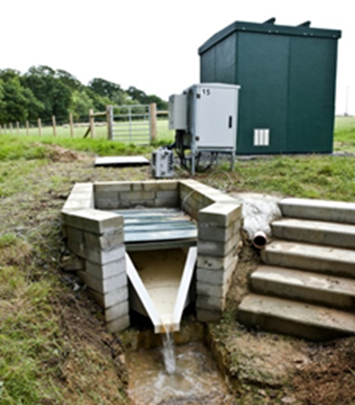
 Picture C: Surface runoff from grassland


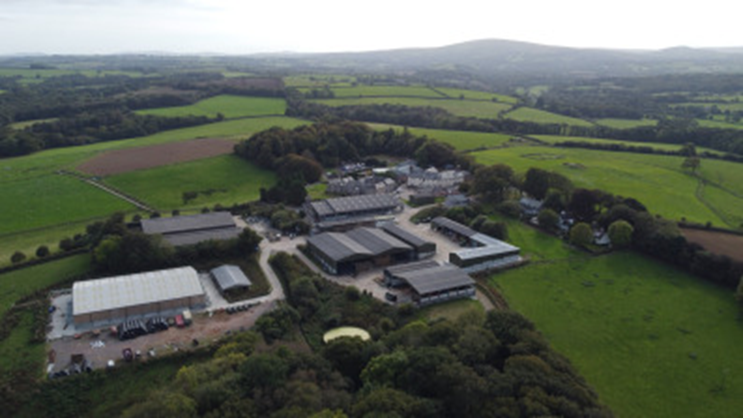


Picture D: Aerial photograph of part of the UTRO
